# BK_Ca_ channel deletion modulates the DNA damage response to electron ultra-high dose rate irradiation in glioblastoma cells

**DOI:** 10.1007/s11060-026-05804-z

**Published:** 2026-09-22

**Authors:** Kamila Maliszewska-Olejniczak, Michał Fryc, Agata Kustra, Katarzyna Wiktorska, Aleksandra Lenartowicz-Gasik, Wojciech Soroka, Jacek Rzadkiewicz, Monika Żochowska, Bogusz Kulawiak, Piotr Bednarczyk

**Affiliations:** 1 Department of Physics and Biophysics, Institute of Biology, Warsaw University of Life Sciences – SGGW, Warsaw, Poland; 2 Laboratory of Intracellular Ion Channels, Nencki Institute of Experimental Biology, Polish Academy of Sciences, Warsaw, Poland; 3 National Centre for Nuclear Research, Otwock-Świerk, Poland

**Keywords:** UHDR irradiation, Radiosensitivity, DNA damage response, BK_Ca_ channel, Ion channels, Glioblastoma

## Abstract

**Purpose:**

Large-conductance Ca^2 +^-activated potassium (BK_Ca_) channels have been implicated in glioblastoma progression and oxidative stress; however, their contribution to the cellular response to ionizing radiation remains poorly understood. Potassium channels represent attractive therapeutic targets because their activity can be modulated pharmacologically using selective inhibitors or genetically by gene silencing (siRNA) or gene knockout (CRISPR/Cas9). Here, we investigated whether genetic inhibition of BK_Ca_ modulates the DNA damage response of human glioblastoma cells following electron ultra-high dose rate (UHDR) irradiation.

**Methods:**

Human U87MG glioblastoma cells and BK_Ca_-knockout cells (U87MG ΔαBK_Ca_) were exposed to an electron UHDR–9 MeV beam with an average dose rate of 150 Gy/s. Clonogenic survival, reactive oxygen species levels, cell-cycle distribution, apoptosis, DNA-DSBs, DNA repair, and expression of selected genes encoding DDR pathways were analyzed.

**Results:**

BK_Ca_ deletion did not significantly alter clonogenic survival following UHDR irradiation. Cells lacking the BK_Ca_ channel exhibited elevated ROS levels, altered cell cycle distribution with G0/G1 accumulation, and enhanced apoptosis. UHDR induced lower γH2AX accumulation and reduced 53BP1 foci formation in U87MG ΔαBK_Ca_ cells, suggesting impaired DNA damage recognition and repair complex assembly. Gene expression analysis revealed a shift toward upregulation of DSBR pathways and downregulation of PARP-dependent SSBR mechanisms.

**Conclusion:**

In conclusion, these findings identify BK_Ca_ as a regulator of the early molecular response of glioblastoma cells to electron UHDR irradiation and provide a basis for further studies investigating BK_Ca_-dependent radiation responses as a target for therapeutic modulation.

**Supplementary Information:**

The online version contains supplementary material available at https://doi.org/10.1007/s11060-026-05804-z.

## Abstract (fr)

BK_Ca_ channel deletion modulates glioblastoma response to UHDR irradiation by altering redox balance and DNA damage signaling. Knockout cells showed increased ROS and apoptosis, reduced γH2AX and 53BP1 recruitment, and a shift toward DSBR pathways.

## Introduction

Glioblastoma multiforme (GBM) is the most aggressive and common primary malignant brain tumor in adults, classified as a grade IV astrocytoma by WHO. It is characterized by rapid growth, invasiveness, and resistance to standard therapies, leading to a poor prognosis with a median survival of 12–15 months despite treatment [1]. Maximal safe surgical resection followed by adjuvant radiotherapy combined with temozolomide (TMZ) constitutes the current standard of care; however, resistance to therapy frequently develops. [1–3]. The poor radiation response of GBM is driven by robust DNA repair mechanisms, apoptosis resistance, glioma stem cell (GSC) survival, autophagy, metabolic plasticity, immune evasion, and microenvironmental factors [4]. Targeting these pathways offers potential avenues to improve radiotherapy outcomes in GBM patients. GBM cells exhibit upregulated DNA damage response (DDR) pathways, such as homologous recombination (HR) and non-homologous end joining (NHEJ), which are crucial for repairing radiation-induced DNA damage [4–7]. The treatment of glioblastoma is particularly challenging because the tumor develops within the highly radiosensitive central nervous system, where radiation-induced injury to surrounding healthy brain tissue limits therapeutic dose escalation. Therefore, novel irradiation strategies capable of improving the therapeutic ratio are highly desirable [8].

FLASH radiotherapy (FLASH-RT) is an emerging modality that delivers ultra-high dose rates (UHDRs) of radiation (>40 Gy/s) in extremely short durations (milliseconds) [9]. It has attracted considerable attention because preclinical studies suggest that it may reduce radiation-induced normal tissue toxicity while maintaining tumor control, a phenomenon known as the FLASH effect. Such tissue-sparing effects could be especially beneficial in glioblastoma therapy, where preservation of normal brain function is of critical importance. However, the biological mechanisms responsible for these differential responses remain incompletely understood [10–13]. FLASH irradiation can be delivered using different radiation modalities, including electrons, protons, and X-rays. In the present study, we employed a UHDR electron beam, as electron-based systems are among the most widely used platforms for preclinical FLASH research due to their accessibility and ability to efficiently deliver ultra-high dose rates [14]. The exact mechanisms underlying the FLASH effect are not fully understood, but it is believed to involve acute oxygen depletion and reduced reactive oxygen species (ROS), which contribute to the sparing of normal tissues [11, 12]. Moreover, UHDR is often associated with decreased levels of DNA damage and/or reduced formation of misrepair products [15]. The precise signaling mechanisms underlying these differential responses remain unclear; however, accumulating evidence suggests a potential role for ROS in mediating these effects.

Glioblastoma cells functionally express various potassium channels, some of which are classified as “oncochannels” due to their role in tumor progression [16–21]. BK_Ca_ channels (large-conductance calcium-activated potassium channels) are overexpressed in glioblastoma models, including established cell lines such as U87MG and T98G, glioblastoma stem-like cells, and primary glioblastoma cells derived from patient biopsies [19, 22, 23]. Among them, a glioma-specific BK_Ca_ splice variant (gBK_Ca_) has been identified and shown to be highly expressed in glioblastoma and glioblastoma stem-like cells [24]. BK_Ca_ channels are functionally upregulated in hypoxic conditions, which are common in the GBM microenvironment. This upregulation enhances the migratory ability of GBM cells and contributes to chemoresistance [19, 22]. In addition, a mitochondrial isoform of BK_Ca_ (mitoBK_Ca_), expressed in glioblastoma cell lines including U87MG, has been implicated in the regulation of mitochondrial function, oxidative stress, and cell survival under hypoxic and cytotoxic stress conditions [25, 26]. BK_Ca_ channels facilitate the migration and invasion of glioblastoma cells by supporting the necessary ion efflux and water extrusion for cell shrinkage during migration [19, 27]. Importantly, although *KCNMA1* is well established as the gene encoding the pore-forming α-subunit of BK_Ca_ channels, recent studies have demonstrated that its products localize to both the plasma membrane and mitochondria in U87MG cells [25]. Furthermore, mitochondrial ROS levels are controlled by the presence of this channel. Loss of the BK_Ca_ channel caused a rise in mitochondrial ROS levels in U87MG ΔαBK_Ca,_ our glioblastoma cell line model [25]. Ionizing radiation can induce ROS formation and oxidative stress, while the magnitude and kinetics of these responses may depend on irradiation type, parameters, oxygen availability, and cellular context [28]. Because BK_Ca_ channels, including their mitochondrial isoform, are involved in the regulation of mitochondrial redox homeostasis, they may influence glioblastoma cell responses to UHDR irradiation. Although our previous work identified BK_Ca_ as a novel regulator of the DDR under oxidative stress conditions following gentoxic agent (particulate matter) exposure, its involvement in DDR triggered by ionizing radiation remains unexplored [29]. Several previous studies have implicated BK_Ca_ channels in the response of glioblastoma cells to ionizing radiation. Steinle et al. demonstrated that conventional X-ray irradiation increased BK_Ca_ channel activity in U87MG and T98G cells, resulting in CaMKII activation and enhanced cell migration [23]. Subsequent work showed that BK_Ca_ channel blockade or knockdown inhibited radiation-induced glioblastoma cell migration and that pharmacological BK_Ca_ targeting reduced tumor cell infiltration in an orthotopic glioblastoma model following fractionated photon irradiation [30]. However, these previous studies were conducted using conventional irradiation and focused primarily on migration, tumor infiltration, or acquired radioresistance. To date, the role of BK_Ca_ in the cellular response of glioblastoma cells to UHDR irradiation remains unexplored. In particular, it is unknown whether BK_Ca_ deficiency affects UHDR-induced oxidative stress, DNA damage, and DNA damage response signaling. Therefore, the present study investigated the consequences of BK_Ca_ deletion for the response of glioblastoma cells to UHDR irradiation, with particular emphasis on ROS generation, DNA damage and early DNA damage response signaling, cell-cycle distribution, and cell death.

## Material and methods

### Cell culture

Human glioblastoma U87MG cells (ATCC, Manassas, VA, USA) were cultured in Dulbecco′s Modified Eagle′s Medium (DMEM) (D5796, Sigma-Aldrich), high glucose with sodium bicarbonate and ʟ-glutamine supplemented with 10% fetal bovine serum (FBS) and 100 U/ml penicillin, and 100 µg/ml streptomycin (37 °C, 5% CO₂, humidified atmosphere). The cell line was authenticated by regular monitoring of cell morphology, and once by a short tandem repeat (STR) analysis in 2013, as previously described [31]. After authentication, the lines were frozen in liquid nitrogen for future in vitro experiments. *Mycoplasma* contamination was monitored on an ongoing basis; the supplementary figure presents the latest test conducted during the experiments for this publication (Supplementary Figure S1). Culture medium was replaced every 2–3 days, and cells were passaged at approximately 70–80% confluence using trypsin–EDTA. Cell morphology was routinely monitored using a CKX53 inverted microscope (Olympus) equipped with CellSens Entry software (v4.2). The U87MG ΔαBK_Ca_ cell line, characterized by the loss of the large conductance calcium-activated potassium channel (BK_Ca_) α-subunit, was generated using CRISPR-Cas9–mediated genome editing. The design of guide RNAs, validation of gene disruption, and functional characterization of the model were previously described by Kulawiak et al. [25]. For experimental procedures, U87MG wt and U87MG ΔαBK_Ca_ cells were subjected to UHDR irradiation (in T-25 flasks and 6-well plates) under the indicated conditions below. Where specified, etoposide (50 µM, 2-hour incubation, Sigma-Aldrich) was used as a positive control for DNA damage (DNA-DSBs) and induced cell cycle perturbation. As a topoisomerase II inhibitor, etoposide induces DNA double-strand breaks and promotes G2/M cell cycle arrest, thereby allowing verification of the sensitivity and proper performance of the BrdU/DAPI assay.

### PCR test for *Mycoplasma* detection

*Mycoplasma* contamination has been shown to alter the microarray gene expression profiles of hundreds of genes in cultured human cells, as demonstrated by Miller et al. [32]. Therefore, *Mycoplasma* detection in U87MG wt and U87MG ΔαBK_Ca_ cell culture supernatants was evaluated using the EZ-PCR Mycoplasma Detection Kit (Biological Industries, Cat. No. 20–700-20) according to the manufacturer’s protocol and our recently published assay [6]. This PCR-based method amplifies conserved sequences within the 16S rRNA gene characteristic of *Mycoplasma* species. The presence of a 270 bp band indicated *Mycoplasma*-specific amplification, while a 357 bp band corresponded to the internal control. Samples displaying both bands were classified as *Mycoplasma*-positive, whereas samples showing only the 357 bp band were considered negative (Supplementary Figure S1).

### Irradiation and dosimetry

Cells were irradiated with an electron beam using an intraoperative linear accelerator, modified to deliver ultra-high dose-rate (FLASH) irradiation [33]. In FLASH mode, the accelerator generates electron beams at 6 and 9 MeV. For the present study, a 9 MeV electron beam with an average dose rate of 150 Gy/s was used. The corresponding UHDR irradiation parameters are listed in Table 1, according to the guidelines published by Böhlen et al. 2024 [34]. Cells were exposed to total absorbed doses of 1.72 ± 0.13 Gy (rounded to a dose of 2 Gy), 3.04 ± 0.09 Gy (3 Gy), and 4.64 ± 0.06 Gy (5 Gy), with 2, 3, and 5 radiation pulses delivered for each dose, respectively, while non-irradiated cells served as the control group. Gafchromic EBT-XD (Ashland Inc., Bridgewater, NJ, USA) radiochromic films were placed beneath the culture flasks to verify the dose delivered to the cell layer. The films were scanned and analyzed 24 h post-irradiation. Dose delivery was additionally monitored using an internal real-time dosimetric system based on inductive sensors, in which the passage of the electron beam induces a voltage in a secondary toroidal winding [35]. The maximum irradiation time did not exceed 40 ms. During all irradiations, cell culture flasks were positioned within an RW3 slab phantom (PTW Freiburg, Germany) at the center of a circular electron beam field with a 100 mm diameter. Irradiations were performed at room temperature under ambient oxygen conditions, with cells positioned at a source-to-surface distance (SSD) of 60 cm. Under these irradiation conditions, the symmetry and homogeneity of the radiation field were previously characterized and confirmed using the same experimental setup [36].

**Table 1:** Beam parameters used for UHDR irradiation

Beam parameter	Value
Radiation type	Electrons
SSD [cm]	60
Nominal energy [MeV]	9
Field size	100 mm diameter
Mean dose rate [Gy/s]	150
Pulse width [µs]	5
Pulse repetition frequency [Hz]	150
Dose per pulse [Gy]	1
Instantaneous dose rate [Gy/s]	2 × 10^5^
Number of pulses	2–5
Total dose [Gy]	1.72–4.64
Irradiation time [ms]	6.68–26.70
Fractionation schedule	Single

### Clonogenic survival assay

The clonogenic cell survival assay was performed as described previously with minor modifications [29, 37–40]. Briefly, approximately 30 min after UHDR irradiation, cells were trypsinized, counted, and seeded at a density of 100 cells per well in 6-well plates containing 2 mL of complete DMEM medium (Sigma-Aldrich, St. Louis, MO, USA). Cells were maintained under standard culture conditions (37 °C, 5% CO₂) for 10 days to allow colony formation. At the end of the incubation period, cultures were gently washed twice with PBS and fixed using 70% ethanol for 10 min at room temperature. Colonies were subsequently stained for 15 min with Coomassie Brilliant Blue solution (0.1% Coomassie Brilliant Blue R-250, 50% methanol, 10% glacial acetic acid, 40% distilled water; Bio-Rad Laboratories, Inc.). Excess dye was removed using Coomassie Blue Destaining solution (Bio-Rad Laboratories, Inc.), and plates were rinsed with tap water and air-dried at room temperature. Colonies consisting of more than 50 cells were scored using a CKX53 inverted microscope (Olympus) equipped with a 10× objective. Plating efficiency (PE) was calculated as the percentage of colonies formed relative to the number of seeded cells: PE = (number of colonies/number of seeded cells) × 100%. The survival fraction (SF) was determined by normalizing the plating efficiency of treated cells to that of untreated controls: SF = (PE treated/PE control) × 100%. All experiments were performed using three independent cell batches.

### ROS analysis

Intracellular oxidative status was assessed using the cell-permeant probe 2‘,7’-dichlorodihydrofluorescein diacetate (H_2_DCFDA), as previously reported [29, 37]. Following deacetylation by intracellular esterases, H_2_DCFDA is oxidized to the fluorescent compound DCF. It detects a broad spectrum of Reactive Oxygen Species (ROS) and reactive nitrogen species (RNS), including hydrogen peroxide (H₂O₂), hydroxyl radicals (•OH), peroxyl radicals (ROO•), peroxynitrite (ONOO^−^), nitrogen dioxide (•NO₂), and hypochlorite (ClO^−^). Thus, this assay reflects the overall intracellular oxidative status but does not directly detect superoxide anions (O₂•^−^) or distinguish between individual reactive species. On the first day, 96-well plates were treated with Bovine Collagen Type I & III (Sigma-Aldrich) diluted 1:10 in deionized water to prevent the cells from detaching during rinsing. Each well was plated with 50,000 cells the following day. On the third day, cells were exposed to 0 and 3 Gy in a minimum of three replicates, along with a positive control of 250 µM H_2_O_2_ (Sigma-Aldrich). After approximately 30 minutes of UHDR (3 Gy) post-irradiation, the cells were incubated with 10 µM DCFDA (Sigma-Aldrich) in HBSS (Sigma-Aldrich) (100 µl per well) after being rinsed with PBS (VWR). A Fluoroskan ASCENT microplate reader (Thermo Fisher Scientific, USA) was used to measure fluorescence at 485 nm and 538 nm. The data were normalized to the control and presented as mean ± SD (*n* = 3). Statistical analysis was performed using a two-way ANOVA followed by Sidak’s multiple-comparison test.

### Flow cytometry acquisition, controls, and gating strategy

Flow cytometric measurements were performed using a BD LSR II flow cytometer (BD Biosciences, San Jose, CA, USA). The resulting flow cytometry data were subsequently analyzed by the authors using BD FACSDiva™ Software v8.0.1. Up to 1 × 10^6^ cells per sample were collected for staining, depending on the respective assay. The number of events retained for downstream analysis was determined after sequential cellular and singlet gating (Fig. S4). A consistent sequential gating strategy was applied across the corresponding experimental conditions and biological replicates. First, the main cellular population was identified based on forward- and side-scatter characteristics (FSC/SSC), thereby excluding events outside the cellular gate, including debris. Sequential scatter-based gating was subsequently applied for singlet discrimination and exclusion of cell aggregates and doublets. Downstream fluorescence-based analyses were performed within the gated singlet population. For cell-cycle analysis, BrdU incorporation and DAPI fluorescence were used to distinguish G0/G1, S, and G2/M populations. Cleaved PARP1-positive cells were identified based on PE fluorescence, whereas γH2AX-positive cells were identified based on Alexa Fluor 647 fluorescence. For Annexin V/PI analysis, single-stained Annexin V and PI controls were used for fluorescence compensation and establishment of the respective fluorescence populations. Cells were classified according to Annexin V and PI fluorescence as viable (Annexin V−/PI−), early apoptotic (Annexin V+/PI−), and late apoptotic/dead (Annexin V+/PI+) cells. The percentages of cells within the respective populations were determined within the gated cellular population. The same gating strategy was consistently applied across the corresponding experimental conditions and biological replicates for each cell line. A representative step-by-step gating hierarchy is provided in Supplementary Figure S4. Appropriate fluorescence controls were included during assay setup. An unstained control was used to assess background fluorescence, and single-stained controls were prepared for the individual markers/fluorochromes used in the respective experiments, including DAPI, cleaved PARP1, γH2AX, Annexin V, and PI. Single-stained controls were used to establish fluorescence compensation where required. Fluorescence-minus-one (FMO) controls were not used.

### Flow cytometry analysis of cell cycle distribution

Cell cycle progression was assessed by BrdU incorporation combined with total DNA staining using our previously described method [29]. U87MG WT and U87MG ΔαBK_Ca_ cells (up to 1 × 10^6^ cells per sample) were subjected to UHDR irradiation. After approximately 30 minutes of UHDR post-irradiation (0 and 3 Gy), cells were pulse-labeled with bromodeoxyuridine (BrdU; final concentration 10 μM; 51–2420KC, BD Pharmingen™) for 60 min at 37 °C. Following labeling, cells were harvested, washed in PBS, and resuspended in 100 μL of BD Cytofix/Cytoperm™ Fixation/Permeabilization Solution (51–2090KE, BD Pharmingen™) for 15 min at room temperature. Cells were washed with 1× BD Perm/Wash™ Buffer (51–2091KE) and incubated with 100 μL of BD Cytofix/Cytoperm™ Plus Permeabilization Buffer (51–2356KC) for 10 min on ice, followed by an additional fixation step (5 min, room temperature). To expose incorporated BrdU, cells were treated with DNase (30 μg per sample; 51–2358KC) for 60 min at 37 °C. After washing, cells were incubated for 20 min at room temperature with PerCP-Cy5.5-conjugated mouse anti-BrdU antibody (5 μL/test; 51–9007682, BD Pharmingen™). Total DNA content was assessed by DAPI staining (1 μg/mL; 51–9007681). Samples were acquired on a BD LSR II flow cytometer (BD Biosciences, San Jose, CA, USA). DAPI fluorescence was collected in linear mode, and cell cycle distribution (G0/G1, S, G2/M) was determined based on DNA content and BrdU incorporation using BD FACSDiva™ Software v8.0.1. Etoposide (ETOP; 50 µM, 2 h incubation) was used as a positive control for DNA damage-induced cell cycle perturbation. As a topoisomerase II inhibitor, etoposide induces DNA double-strand breaks and promotes G2/M cell cycle arrest. All experiments were performed using three independent cell batches. For cell cycle distribution analysis, two-way ANOVA with Sidak’s multiple comparisons test was applied. A *p*-value ≤ 0.05 was considered statistically significant (*p ≤ 0.05, **p ≤ 0.01, ***p ≤ 0.001).

### Annexin V-FITC apoptosis assay

The number of U87MG wt and U87MG ΔαBK_Ca_ cells undergoing apoptosis and/or necrosis following UHDR irradiation was quantified using the eBioscience™ Annexin V Apoptosis Detection Kit FITC (Invitrogen, Thermo Fisher Scientific, Cat. No. 88–8005-72), according to the manufacturer’s instructions. Briefly, 5 × 10^5^ cells were seeded in T-25 culture flasks 24 h before irradiation. On the following day, cells were exposed to a single dose of 3 Gy UHDR irradiation or left non-irradiated as controls. 24 hours after irradiation, cells were collected by trypsinization and washed once with PBS, followed by one wash with 1× Binding Buffer. The cells were then resuspended in 100 μL of 1× Binding Buffer. For staining, 100 μL of the cell suspension was mixed with 5 μL of Annexin V-FITC. Samples were incubated for 15 min at room temperature in the dark. The cells were subsequently washed with 1× Binding Buffer and resuspended in 200 μL of 1× Binding Buffer, after which 5 μL of propidium iodide staining solution was added. Samples were kept protected from light and analyzed by flow cytometry within 2 h using an LSRFortessa flow cytometer (BD Biosciences, San Jose, CA, USA). Data were analyzed using FACSDiva software (BD Biosciences). For flow cytometric analysis, appropriate controls were included for each experiment: unstained cells, cells stained with PI only, and cells stained with Annexin V-FITC only. Cells negative for both Annexin V-FITC and PI were classified as viable, Annexin V-FITC-positive/PI-negative cells as early apoptotic, Annexin V-FITC-positive/PI-positive cells as late apoptotic, and Annexin V-FITC-negative/PI-positive cells as necrotic. Each experiment was repeated three times using independent cell cultures (*n* = 3). Statistical analysis was performed using two-way ANOVA followed by Tukey’s multiple-comparison test. A *p*-value ≤ 0.05 was considered statistically significant (*p ≤ 0.05, **p ≤ 0.01, ***p ≤ 0.001).

### Flow cytometry quantification of PARP1 cleavage

PARP1 cleavage (Asp214) was measured using our previously described method [29]. After approximately 30 minutes post-irradiation (0 and 3 Gy), up to 1 × 10^6^ cells per sample were collected, washed in PBS, and fixed/permeabilized using BD Cytofix/Cytoperm™ Fixation/Permeabilization Solution (51–2090KE) for 20 min at room temperature. After washing with 1× BD Perm/Wash™ Buffer, cells were incubated with BD Cytofix/Cytoperm™ Plus Permeabilization Buffer for 10 min on ice, then refixed for 5 min. Cells were then incubated for 20 min at room temperature with PE-conjugated mouse anti-cleaved PARP (Asp214) antibody (5 μL/test; 51–9007684, BD Pharmingen™). After washing, samples were resuspended in staining buffer and analyzed by flow cytometry using a BD LSR II instrument. Cleaved PARP-positive cells were quantified as a percentage of the total gated cell population. Etoposide (ETOP; 50 µM, 2 h incubation) was used as a positive control for apoptosis induction, as it induces DNA double-strand breaks and activates caspase-dependent PARP cleavage, thereby allowing verification of the sensitivity and proper performance of the assay. Experiments were performed using three independent cell batches. Statistical analysis was performed using one-way ANOVA followed by Tukey’s multiple-comparison test. A *p*-value ≤ 0.05 was considered statistically significant (*p ≤ 0.05, **p ≤ 0.01, ***p ≤ 0.001).

### Alkaline comet assay

The alkaline comet assay was performed according to our previously published procedure [37]. Briefly, one day before irradiation, U87MG WT and U87MG ΔαBK_Ca_ cells were seeded in 6-well plates at a density of 0.3 × 10^6^ cells per well. On the following day, cells were irradiated with 3 Gy. As a positive control for the comet assay, cells were treated with 50 µM etoposide for 2 h. Approximately 30–60 min after irradiation, cells were collected, washed, and resuspended in ice-cold PBS (VWR). A 50 µL aliquot of the cell suspension was mixed with 500 µL of molten agarose (Bio-Techne, R&D Systems), and 50 µL of the resulting cell–agarose mixture was applied to each well of a CometSlide (Bio-Techne, R&D Systems). The slides were incubated in the dark at 2–8 °C for 10 min to allow the agarose to solidify and were subsequently immersed in lysis solution (Bio-techne, R&D Systems) for 60 min at 4 °C. DNA was then allowed to unwind for 20 min at room temperature in the dark in an alkaline solution (pH > 13; 0.4 g NaOH, 250 µL of 200 mM EDTA, pH 10, and 49.75 mL H₂O). Electrophoresis was performed under alkaline conditions (pH 13) in a cooled Comet Assay Tank (Cleaver Scientific) using electrophoresis buffer containing 8 g NaOH and 2 mL of 500 mM EDTA in a final volume of 1 L. Following electrophoresis, the slides were washed, fixed in 70% ethanol (POCH), and allowed to dry completely. DNA was stained with 100 µL of diluted SYBR Safe solution (Invitrogen) for 30 min in the dark. After drying, the slides were analyzed under 40x objective using an Olympus FluoView FV1200 confocal laser scanning microscope at excitation/emission wavelengths of 502/530 nm. Comet images were analyzed using OpenComet v1.3.1, and DNA damage was quantified as the percentage of DNA in the comet head (% head DNA) and comet tail (% tail DNA). At least 50 comets per experimental condition were analyzed in each biological replicate. Three independent experiments were performed (*n* = 3). Statistical analysis was performed using one-way ANOVA followed by Tukey’s multiple-comparison test. A *p*-value ≤ 0.05 was considered statistically significant (*p ≤ 0.05, **p ≤ 0.01, ***p ≤ 0.001).

### Flow cytometric analysis of phosphorylated histone γH2AX

Flow cytometry was used to quantify IR-induced phosphorylation of histone H2AX at serine 139 (γH2AX), as previously performed in our studies [29]. U87MG cells (1 × 10^6^ per sample) were harvested after approximately 30 minutes of UHDR (0 and 3 Gy) post-irradiation and fixed with BD Cytofix/Cytoperm™ Fixation/Permeabilization Solution for 20 min at room temperature. Cells were washed and further permeabilized using BD Cytofix/Cytoperm™ Plus Permeabilization Buffer for 10 min on ice. Following permeabilization, cells were incubated with Alexa Fluor™ 647-conjugated mouse anti-H2AX (pS139) antibody (5 μL/test; 51–9007683, BD Pharmingen™) for 20 min at room temperature in the dark. After washing with 1× BD Perm/Wash™ Buffer, cells were resuspended in staining buffer and analyzed by flow cytometry. Etoposide (ETOP; 50 µM, 2 h incubation) was used as a positive control for DNA damage induction, as it induces DNA double-strand breaks and robust H2AX phosphorylation, thereby enabling verification of the sensitivity and proper performance of the γH2AX assay. Data acquisition was performed using a BD LSR II flow cytometer and analyzed with BD FACSDiva™ Software v8.0.1. All measurements were obtained from three independent cell batches. Statistical analysis was performed using one-way ANOVA followed by Tukey’s multiple-comparison test. A *p*-value ≤ 0.05 was considered statistically significant (*p ≤ 0.05, **p ≤ 0.01, ***p ≤ 0.001).

### Immunofluorescence staining and visualization

For immunofluorescence analysis, U87MG WT and U87MG ΔαBK_Ca_ cells were seeded 1 day before the experiment at approximately 3 × 10^5^ cells per well on glass coverslips in 6-well plates, as previously described and appropriately modified [41]. 30 min after UHDR irradiation (0 and 3 Gy), the culture medium was removed, and the cells were gently washed with PBS. Cells were fixed in 4% paraformaldehyde (Merck) for 10 min at room temperature, followed by two washes in PBS (5 min each). Permeabilization was performed using 0.1% Triton X-100 in PBS for 10 min at room temperature. Cells were subsequently washed in PBS containing 0.05% Triton X-100. Non-specific binding sites were blocked with 1% BSA in PBS supplemented with 0.05% Triton X-100 for 1 h at room temperature in a humidified dark chamber. After a single wash (PBS + 0.05% Triton X-100), cells were incubated overnight at 4 °C with primary antibody diluted in 0.1% BSA in PBS containing 0.05% Triton X-100. 53BP1 foci were detected using rabbit anti-53BP1 antibody (EPR2172(2), Abcam, ab175933; 1:200 dilution). Following primary antibody incubation, cells were washed (3 × 5 min) with PBS containing 0.05% Triton X-100. Secondary antibody incubation was performed for 1 h at room temperature using Goat Anti-Rabbit IgG H&L conjugated to Alexa Fluor® 594 (Abcam, ab150080; 1:400 dilution) diluted in 0.1% BSA in PBS containing 0.05% Triton X-100. After secondary antibody incubation, cells were washed three times in PBS. Coverslips were mounted onto glass slides using ProLong™ Gold Antifade Mountant with DAPI (Invitrogen, P36935) to counterstain nuclei and prevent photobleaching. Slides were sealed with transparent nail polish and stored at 4 °C, protected from light until imaging. Etoposide (ETOP; 50 µM, 2 h incubation) was used as a positive control for DNA damage induction, as it promotes the formation of DNA double-strand breaks and subsequent recruitment of 53BP1 to DNA damage foci, thereby confirming the sensitivity of the immunofluorescence assay. Images were acquired using a Leica TCS SP5 confocal microscope (Leica Microsystems). Alexa Fluor® 594 was excited using a 561 nm laser line, and emission was collected in the 590–620 nm range. DAPI was excited using a UV laser. Images were obtained using a 63× oil-immersion objective (under identical acquisition parameters for all experimental groups).

### RNA isolation and qPCR

Total RNA was extracted from U87MG WT and U87MG ΔαBK_Ca_ cells seeded 24 h before the experiment in T-25 culture flasks. RNA isolation was performed after 30 min of UHDR irradiation (0 and 3 Gy), using the RNeasy Mini Kit (Qiagen, TX, USA) according to the manufacturer’s protocol, including DNase I (Qiagen, TX, USA) treatment to eliminate potential genomic DNA contamination, as described previously [6, 29, 37]. RNA quantity and purity were assessed spectrophotometrically using µDrop™ Duo Plates with a Multiskan SkyHigh microplate reader (Thermo Scientific, DE, USA). Samples exhibiting appropriate A260/A280 ratios were used for downstream applications. For cDNA synthesis, 1 μg of total RNA was reverse-transcribed using the iScript™ cDNA Synthesis Kit (Bio-Rad) containing RNase H+ MMLV reverse transcriptase, according to the manufacturer’s recommendations. mRNA profiling of selected genes involved in DNA damage response and repair pathways (listed alphabetically: *ATM*, *ATR*, *BRCA1*, *H2AFX*, *LIG1*, *LIG3*, *OGG1*, *PARP1*, *PARP2*, *PARP3*, *RAD51*, *TP53BP1*, *XRCC1*, *XRCC4,* listed in Supplementary Table 1) was performed using a custom-designed PrimePCR™ SYBR® Green assay plate (Bio-Rad Laboratories, Inc.) in accordance with the manufacturer’s recommendations and as described previously [6, 29, 37]. *ACTB* (beta-actin) was used as the endogenous reference gene for normalization. Each qPCR reaction contained 1 µL of synthesized cDNA, iTaq™ Universal SYBR® Green Supermix (Bio-Rad), and pre-dried 20× PrimePCR™ assay primers (unique Bio-Rad PrimePCR™Assay IDs were listed in Supplementary Table S1) supplied in 96-well format, prepared according to the SYBR® Green reaction setup protocol for custom plates. Amplification was carried out using a CFX Opus 96 Real-Time PCR System (Bio-Rad). Relative mRNA levels were determined using CFX Manager™ Software (Bio-Rad). Quantification of transcript levels was performed using the 2^−ΔΔCt method. The data represent results obtained from three independent cell batches. Differences between groups were assessed using one-way analysis of variance (ANOVA). A *p*-value ≤ 0.05 was considered statistically significant (*p ≤ 0.05, **p ≤ 0.01, ***p ≤ 0.001).

### Western blot analysis

Western blot analysis was performed as described previously [29]. Briefly, after approximately 30 minutes post-irradiation (0 and 3 Gy), U87MG WT and U87MG ΔαBK_Ca_ cells cultured in T-25 flasks were harvested by scraping in cold PBS, washed with cold PBS, and lysed on ice in RIPA buffer supplemented with protease inhibitors. Cell disruption was enhanced using a glass homogenizer (Wheaton Tight, USA). Lysates were clarified by centrifugation, and total protein concentration was determined using the Bradford assay. Equal amounts of protein (40 µg per sample) were separated by electrophoresis on 10% Tris–Tricine SDS-PAGE gels and transferred onto PVDF membranes (Bio-Rad Laboratories, Inc.). Membranes were blocked for 1 h at room temperature in TBST buffer (50 mM Tris, 150 mM NaCl, 0.2% Tween-20, pH 8.4) containing 10% non-fat milk. Membranes were then incubated with primary antibodies directed against DNA damage response–related proteins, including PARP1 (Anti-PARP Polyclonal Antibody, Thermo Fisher Scientific, USA, PA5-34803, 1:1000) and 53BP1 (R&D Systems, AF1877, 1:200), as well as β-actin (Anti-β Actin antibody, Abcam ab49900, diluted 1:1000, incubated 2 h) as a loading control. Antibodies were diluted in TBST containing 5% non-fat milk and incubated under optimized conditions. After washing, membranes were incubated with appropriate HRP-conjugated secondary antibodies (Anti-Rb, 1:5000). Protein bands were visualized using enhanced chemiluminescence (ECL Prime, Amersham, RPN 2236). Band intensities were quantified using ImageJ software (v1.54p) after background subtraction and normalized to the corresponding β-actin levels. The ratios were calculated from three independent biological experiments (*n* = 3) and are presented as mean ± SD (Fig. 3 and Fig. S3 -western blot original files).

### Statistical analysis

All experiments were performed using three independent biological replicates unless otherwise indicated. Data are presented as mean ± SD. Statistical analyses were conducted using GraphPad Prism version 8 (GraphPad Software, San Diego, CA, USA). Clonogenic survival data were analyzed using two-way repeated-measures ANOVA followed by Sidak’s multiple-comparison test. ROS and cell-cycle distribution data were analyzed using two-way ANOVA followed by Sidak’s multiple-comparison test. For most other experiments, including Annexin V/PI staining, cleaved PARP1 analysis, γH2AX accumulation, 53BP1 foci formation, and Western blot densitometry, differences between groups were assessed using one-way ANOVA followed by Tukey’s multiple-comparison test. Comet assay data were analyzed using one-way ANOVA followed by Dunnett’s multiple-comparison test. A *p*-value < 0.05 was considered statistically significant. Statistical significance is indicated as *p < 0.05, **p < 0.01, and ***p < 0.001; ns, not significant.

## Results

### Clonogenic survival analysis of U87MG wt and U87MG ΔαBK_Ca_ cells under UHDR irradiation

**Fig. 1:**
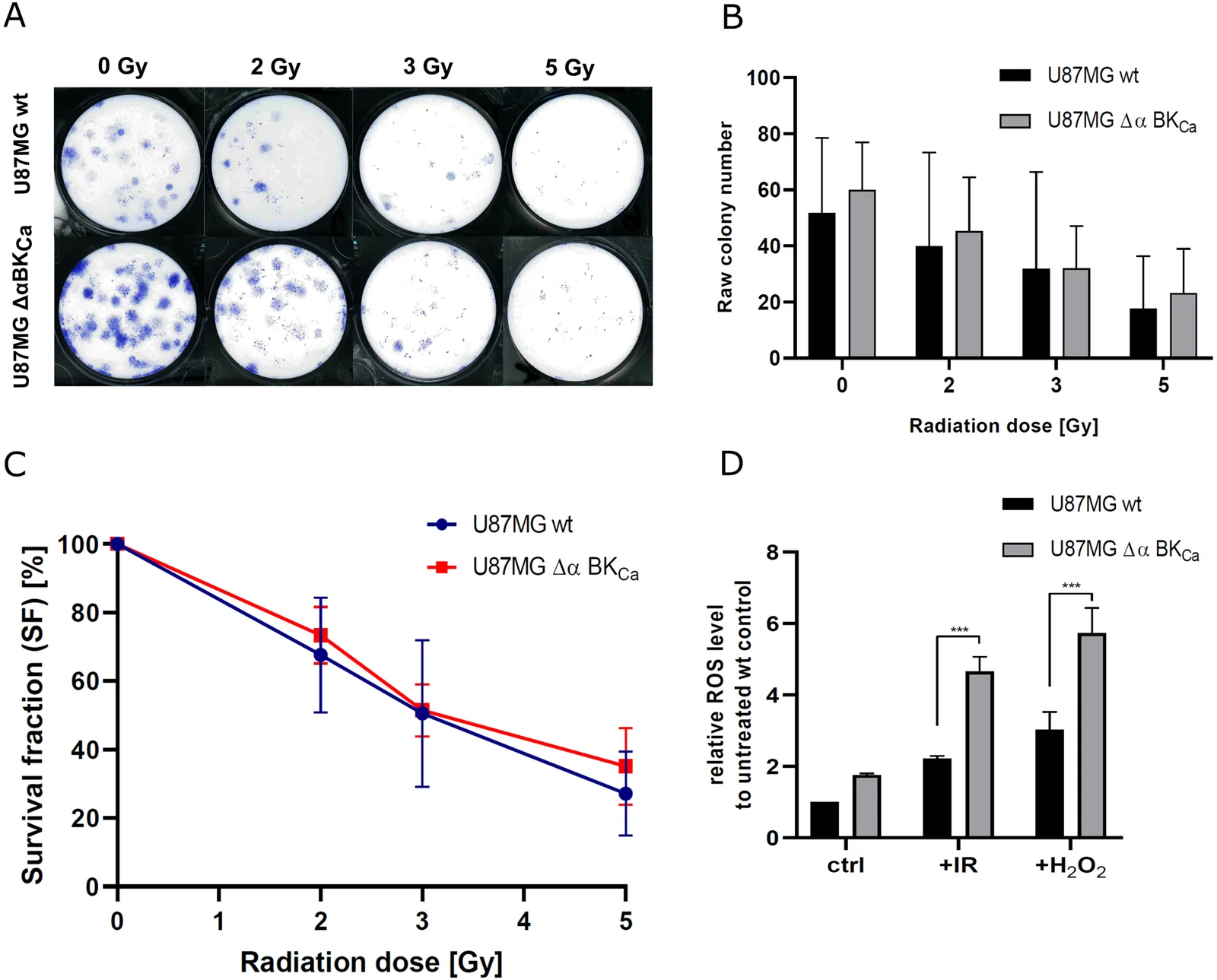
Clonogenic survival and intracellular ROS levels in U87MG cells following UHDR irradiation. A: Representative images of colonies formed by U87MG wt and U87MG ΔαBK_Ca_ cells following irradiation with 0, 2, 3, or 5 Gy. B: raw colony numbers for U87MG wt and U87MG ΔαBK_Ca_ cells following irradiation with 0, 2, 3, or 5 Gy. C: survival fraction of U87MG wt and U87MG ΔαBK_Ca_ cells following increasing doses of UHDR irradiation, expressed as a percentage relative to the corresponding non-irradiated control. Data are presented as mean ± SD from three independent experiments. D: intracellular ROS levels in U87MG wt and U87MG ΔαBK_Ca_ cells under control conditions, following 3 Gy UHDR irradiation (+IR), or treatment with 250 µM hydrogen peroxide (+H₂O₂). ROS levels were determined using a fluorescence-based assay and are expressed relative to the U87MG wt control, which was set to 1. Data are presented as mean ± SD from at least three independent experiments. Clonogenic survival data were analyzed using two-way repeated-measures ANOVA followed by Sidak’s multiple-comparison test, and ROS data were analyzed using two-way ANOVA followed by Sidak’s multiple-comparison test. Statistical significance is indicated as *p < 0.05, **p < 0.01, and ***p < 0.001

Clonogenic survival assays were performed to assess the long-term proliferative capacity of U87MG wt and U87MG ΔαBK_Ca_ cells following UHDR (Fig. 1). Representative images of colony formation demonstrate a progressive reduction in colony number with increasing radiation dose in both cell lines (Fig. 1A). Quantitative analysis of the colonies and survival fraction confirmed a dose-dependent decrease in clonogenic survival in both cell lines (Fig. 1B and C). In U87MG wt cells, the survival fraction decreased from 100% in non-irradiated controls to 67.59 ± 29.03% at 2 Gy, 50.51 ± 37.14% at 3 Gy, and 27.10 ± 21.20% at 5 Gy. Similarly, U87MG ΔαBK_Ca_ cells showed survival fractions of 73.37 ± 14.24% at 2 Gy, 51.43 ± 13.20% at 3 Gy, and 35.09 ± 19.44% at 5 Gy. Although the mean survival fractions were numerically higher in U87MG ΔαBK_Ca_ cells at 2 and 5 Gy, no significant genotype-dependent differences were observed at any of the tested radiation doses. Based on these observations, a radiation dose of 3 Gy was selected for mechanistic analyses, as it produced a clear reduction in clonogenic survival while maintaining sufficient viable cells for downstream assays investigating DNA damage response pathways.

### BK_Ca_ deficiency enhances UHDR irradiation-induced oxidative stress in glioblastoma cells

To assess the total level of reactive oxygen species (ROS) in U87MG cells following 3 Gy UHDR irradiation (IR), intracellular ROS levels were measured using a fluorescence-based assay. Figure 1C shows ROS levels expressed as arbitrary units (a.u.). Under basal conditions, no significant differences in ROS levels were observed between U87MG wt and U87MG ΔαBK_Ca_ cells (1 vs 1.76 ± 0.04). Following irradiation, a significant increase in ROS levels was detected in ΔαBK_Ca_ cells (4.66 ± 0.4) compared with wt cells (2.21 ± 0.08; ***p ≤ 0.001), indicating enhanced oxidative stress in BK_Ca_-deficient cells. Treatment with H₂O₂ (positive control) further elevated ROS levels in both cell lines; however, U87MG ΔαBK_Ca_ cells exhibited significantly higher ROS accumulation (5.73 ± 0.71) compared with wt cells (3.02 ± 0.5; ***p ≤ 0.001).

### Loss of BK_Ca_ modulates UHDR-induced cell cycle changes

**Fig. 2:**
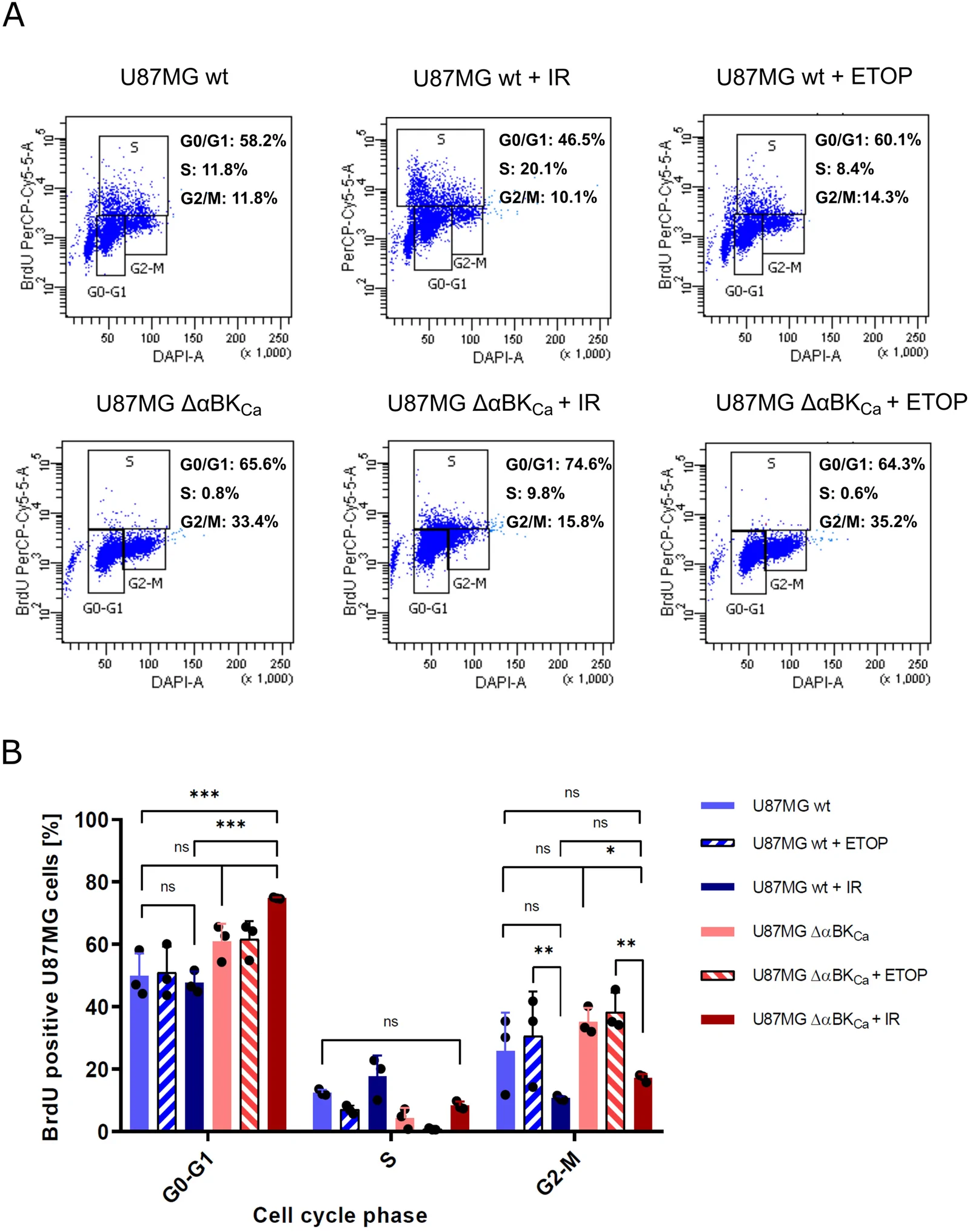
Cell cycle analysis of U87MG cells following UHDR irradiation. A: Representative flow cytometry plots showing cell cycle distribution determined by BrdU incorporation and DAPI staining in U87MG wt and U87MG ΔαBK_Ca_ cells under control conditions and after UHDR (IR, 3 Gy) and 50 µM etoposide (ETOP) treatment as a positive control of G2/M cell cycle arrest. The cells were labeled with 10 µM BrdU for 1 h and stained with BrdU PerCP-Cy5.5 antibody. Cells were classified into G0/G1, S, and G2/M phases based on BrdU incorporation and DNA content. B: quantification of the percentage of cells in G0/G1, S, and G2/M phases. Data represent mean ± SD from three independent experiments. Statistical analysis was performed using two-way ANOVA followed by Sidak’s multiple comparisons test. Statistical significance is indicated as *p ≤ 0.05, **p ≤ 0.01, ***p ≤ 0.001; ns, not significant

To further investigate the effect of BK_Ca_ deletion and UHDR irradiation on glioblastoma cell proliferation, cell cycle distribution was analyzed using BrdU incorporation combined with flow cytometry. This approach enables precise identification of cells undergoing DNA synthesis and assessment of cell cycle phase alterations following exposure. Cell cycle distribution was assessed by BrdU incorporation and DAPI staining in U87MG wt and U87MG ΔαBK_Ca_ cells under basal conditions and following irradiation (IR) or etoposide (ETOP) treatment (Fig. 2A–B). Etoposide (50 µM) was used as a positive control for DNA damage-induced cell cycle perturbation. As a topoisomerase II inhibitor, etoposide induces DNA double-strand breaks and promotes G2/M cell cycle arrest, thereby allowing verification of the sensitivity and proper performance of the BrdU/DAPI assay. Under basal conditions, no significant differences were observed. Following 30 min of UHDR irradiation (IR, 3 Gy), wt cells showed a redistribution characterized by 47.7 ± 3.5% in G0/G1, 17.7 ± 6.8% in S phase, and 10.7 ± 0.7% in G2/M. In contrast, irradiated U87MG ΔαBK_Ca_ cells accumulated predominantly in G0/G1 (74.8 ± 0.3% vs 47.7 ± 3.5%; ***p < 0.001)), with reduced fractions in S phase (8.3 ± 1.3% vs 17.7 ± 6.8% (ns, *p* = 0.6715)) and G2/M (17.2 ± 1.2% vs 10.7 ± 0.7% (ns, *p* = 0.9671)). Under ETOP exposure, the G2/M fraction reached 38.3 ± 6.2% in ΔαBK_Ca_ cells and 30.5 ± 14.3% in wt cells, which were significantly higher compared with IR-treated cells in both ΔαBK_Ca_ (**p ≤ 0.01) and wt cells (**p ≤ 0.01). The G2/M fraction was higher in ΔαBK_Ca_ cells compared with ΔαBK_Ca_ + IR cells (35.2 ± 4.5% vs 17.2 ± 1.2% (*p ≤ 0.05)).

### UHDR irradiation affected cell viability and induced early apoptotic cell death in both U87MG wt and U87MG ΔαBK_Ca_ cells

The percentage of viable cells decreased from 88.57 ± 1.18% to 81.07 ± 5.35% in U87MG wt cells (***p < 0.001) and from 88.10 ± 1.85% to 78.90 ± 1.40% in U87MG ΔαBK_Ca_ cells (***p < 0.001). No significant differences in cell viability were observed between U87MG wt and U87MG ΔαBK_Ca_ cells under control conditions (*p* = 0.9922) or following UHDR irradiation (*p* = 0.5714). Early apoptosis increased from 10.23 ± 1.27% to 16.40 ± 5.11% in U87MG wt cells (**p < 0.01) and from 8.67 ± 1.46% to 16.43 ± 1.15% in U87MG ΔαBK_Ca_ cells (***p < 0.001). No significant differences in early apoptosis were observed between the two cell lines under control conditions (*p* = 0.7848) or following UHDR irradiation (*p* > 0.9999). Late apoptosis increased numerically from 0.37 ± 0.06% to 0.73 ± 0.15% in U87MG wt cells (*p* = 0.9962) and from 0.80 ± 0.20% to 1.50 ± 0.26% in U87MG ΔαBK_Ca_ cells (*p* = 0.9748); however, neither change was statistically significant. No significant differences in late apoptosis were observed between U87MG wt and U87MG ΔαBK_Ca_ cells under control conditions (*p* = 0.9938) or following irradiation (*p* = 0.9674). Necrosis increased numerically from 0.87 ± 0.15% to 1.80 ± 0.17% in U87MG wt cells (*p* = 0.9435) and from 2.43 ± 0.21% to 3.17 ± 0.42% in U87MG ΔαBK_Ca_ cells (*p* = 0.9712), but these changes were not statistically significant. No significant genotype-dependent differences in necrosis were detected under control conditions (*p* = 0.7848) or following UHDR irradiation (*p* = 0.8454).

### UHDR irradiation increases PARP1 cleavage in BK_Ca_-deficient cells

**Fig. 3:**
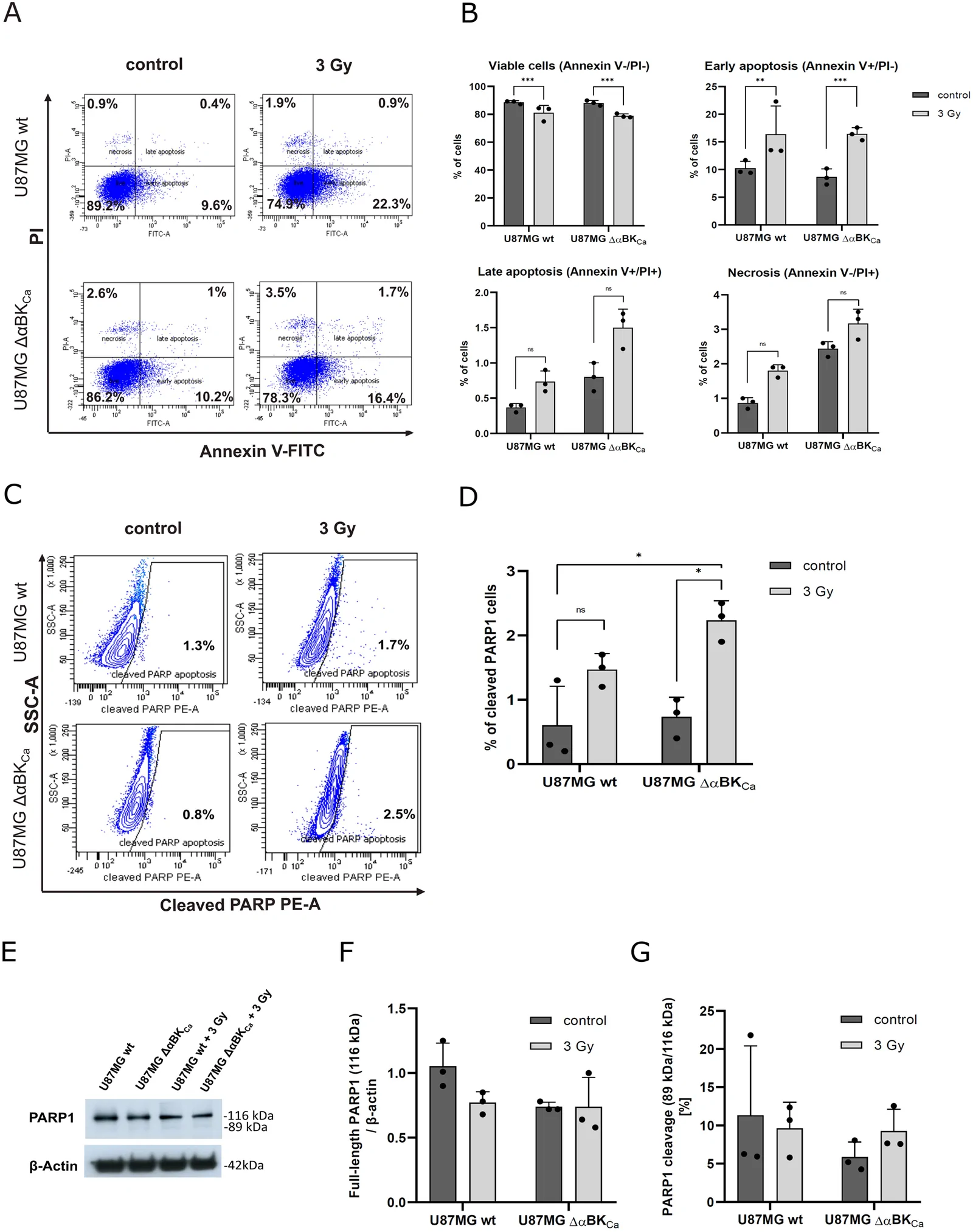
Apoptotic response and PARP1 cleavage in U87MG cells following UHDR irradiation. A: Representative flow cytometry plots of Annexin V-FITC/PI staining in U87MG wt and U87MG ΔαBK_Ca_ cells under control conditions and following 3 Gy UHDR irradiation. Cells were classified as viable (Annexin V−/PI−), early apoptotic (Annexin V+/PI−), late apoptotic (Annexin V+/PI+), or necrotic (Annexin V−/PI+). B: quantification of viable, early apoptotic, late apoptotic, and necrotic cell populations. Data are presented as mean ± SD from three independent experiments. C: Representative flow cytometry plots showing cleaved PARP1-positive cells in U87MG wt and U87MG ΔαBK_Ca_ cells under control conditions and following 3 Gy UHDR irradiation. Cleaved PARP1 was detected using a PE-conjugated antibody recognizing the Asp214-cleaved form of PARP1. D: quantification of cleaved PARP1-positive cells. Data are presented as mean ± SD from three independent experiments. E: Representative Western blot analysis of full-length PARP1 (~116 kDa) and cleaved PARP1 (~89 kDa) in U87MG wt and U87MG ΔαBK_Ca_ cells under control conditions and following 3 Gy UHDR irradiation. β-actin (~42 kDa) was used as a loading control. F: densitometric quantification of full-length PARP1 normalized to β-actin. G: PARP1 cleavage expressed as the cleaved-to-full-length PARP1 ratio (89/116 kDa) × 100%. Western blot quantification was performed from three independent biological experiments, and data are presented as mean ± SD. Statistical analysis was performed using one-way ANOVA followed by Tukey’s multiple-comparison test. Statistical significance is indicated as *p < 0.05, **p < 0.01, and ***p < 0.001; ns, not significant

To determine whether BK_Ca_ loss influences the early apoptotic response to irradiation, PARP1 cleavage was assessed in U87MG wt and U87MG ΔαBK_Ca_ cells 30 min after 3 Gy UHDR irradiation. PARP1 is cleaved by caspases-3 and −7 during apoptosis, generating an 89-kDa fragment that is widely used as a marker of apoptotic signaling. Flow cytometric analysis showed a low percentage of cleaved PARP1-positive cells under basal conditions in both U87MG wt (0.60 ± 0.61%) and U87MG ΔαBK_Ca_ cells (0.73 ± 0.31%; *p* = 0.9744). Following UHDR irradiation, the percentage of cleaved PARP1-positive cells increased to 1.47 ± 0.25% in U87MG wt cells, although this change was not statistically significant (*p* = 0.1021). In contrast, UHDR irradiation significantly increased the cleaved PARP1-positive fraction in U87MG ΔαBK_Ca_ cells to 2.23 ± 0.31% (**p < 0.01). However, no significant difference was detected between irradiated U87MG wt and U87MG ΔαBK_Ca_ cells (*p* = 0.1576) (Fig. 3C–D). PARP1 protein expression and cleavage were additionally assessed by Western blotting in three independent biological experiments (Fig. 3E–G). Full-length PARP1 levels normalized to β-actin were 1.05 ± 0.18 and 0.77 ± 0.09 in control and irradiated U87MG wt cells, respectively (*p* = 0.1804), and 0.74 ± 0.03 and 0.74 ± 0.23 in control and irradiated U87MG ΔαBK_Ca_ cells, respectively (*p* = 1.0000). No significant difference in full-length PARP1 levels was observed between the irradiated cell lines (*p* = 0.9946). PARP1 cleavage, expressed as the cleaved-to-full-length PARP1 ratio (89/116 kDa) × 100, was 11.33 ± 9.08% and 9.62 ± 3.41% in control and irradiated U87MG wt cells, respectively (*p* = 0.9760), and 5.84 ± 1.98% and 9.26 ± 2.85% in control and irradiated U87MG ΔαBK_Ca_ cells, respectively (*p* = 0.8470). No significant difference in the PARP1 cleavage ratio was observed between irradiated U87MG wt and U87MG ΔαBK_Ca_ cells (*p* = 0.9997).

### UHDR irradiation induces DNA damage in U87MG wt and U87MG ΔαBK_Ca_ cells

**Fig. 4:**
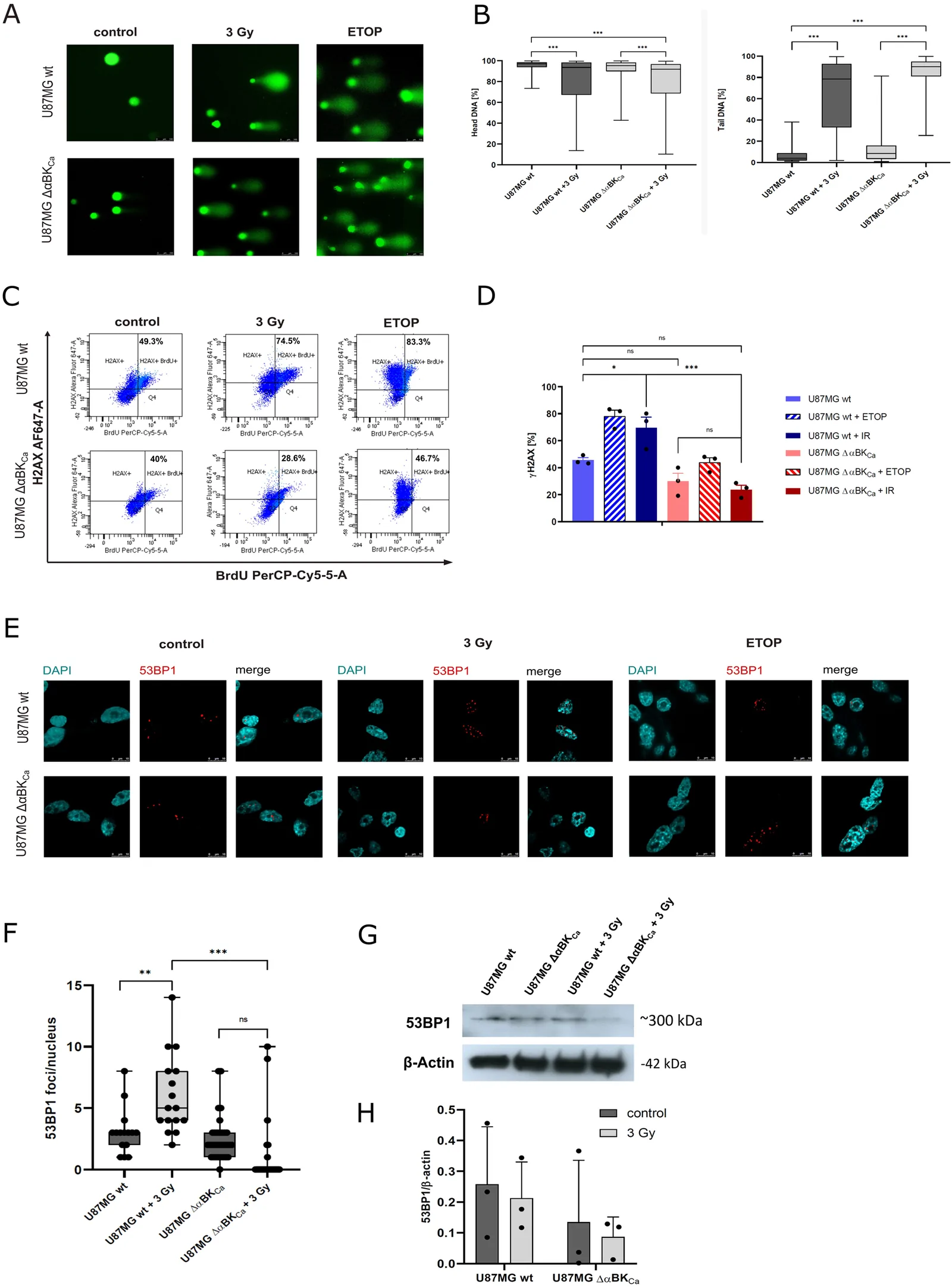
DNA damage and DNA damage response signaling in U87MG cells following UHDR irradiation. A: Representative images from the alkaline comet assay in U87MG wt and U87MG ΔαBK_Ca_ cells under control conditions, following 3 Gy UHDR irradiation. ETOP was used as a positive control. B: quantification of comet assay images was performed using OpenComet v1.3.1 software and expressed as the percentage of DNA in the comet head (head DNA, %) and tail (tail DNA, %). Data are presented as box plots and are based on the analysis of at least 50 comets per condition in each of three independent biological experiments (*n* = 3). Statistical analysis was performed using one-way ANOVA followed by Dunnett’s multiple-comparison test. C: Representative flow cytometry plots showing γH2AX (H2AX pS139, Alexa Fluor 647) in relation to BrdU incorporation (PerCP-Cy5.5) in U87MG wt and U87MG ΔαBK_Ca_ cells under control conditions, following 3 Gy UHDR irradiation, and after treatment with 50 µM ETOP. Percentages indicate the γH2AX-positive cell population in the representative experiments. D: quantification of γH2AX-positive cells determined by flow cytometry. Data are presented as mean ± SD from three independent experiments (*n* = 3). ETOP was used as a positive control. Statistical analysis was performed using one-way ANOVA followed by Tukey’s multiple-comparison test. E: Representative immunofluorescence images of 53BP1 foci in U87MG wt and U87MG ΔαBK_Ca_ cells under control conditions, following 3 Gy UHDR irradiation, and after treatment with 50 µM ETOP. Nuclei were counterstained with DAPI (cyan), and 53BP1 foci are shown in red (Alexa Fluor 594). Scale bar: 10 µm. F: quantification of 53BP1 foci per nucleus. At least 15 nuclei were analyzed per condition. Data are presented as mean ± SD. Statistical analysis was performed using one-way ANOVA followed by Tukey’s multiple-comparison test. G: Representative Western blot analysis of 53BP1 protein levels in U87MG wt and U87MG ΔαBK_Ca_ cells under control conditions and following 3 Gy UHDR irradiation. 53BP1 was detected at ~ 300 kDa, and β-actin (~42 kDa) was used as a loading control. H: densitometric quantification of 53BP1 protein levels normalized to β-actin. Quantification was performed from three independent biological experiments (*n* = 3), and data are presented as mean ± SD. Statistical analysis was performed using one-way ANOVA followed by Tukey’s multiple-comparison test. Statistical significance is indicated as *p ≤ 0.05, **p ≤ 0.01, and ***p ≤ 0.001; ns, not significant

Since UHDR irradiation induced oxidative stress, we next investigated whether this response was accompanied by DNA damage. DNA strand damage was assessed using the alkaline comet assay in U87MG wt and U87MG ΔαBK_Ca_ cells following 3 Gy UHDR irradiation. Etoposide (ETOP) was included as a positive control to verify proper comet formation and electrophoretic DNA migration under the applied assay conditions. The expected pronounced comet-tail formation observed in ETOP-treated cells is shown in the representative images (Fig. 4A), confirming the appropriate performance of the assay. Representative images of irradiated cells also showed pronounced DNA migration and comet-tail formation compared with non-irradiated cells (Fig. 4A). Quantitative analysis of DNA distribution between the comet head and tail confirmed increased DNA damage following UHDR irradiation (Fig. 4B). Head DNA decreased from 95.00 ± 5.56% in non-irradiated U87MG wt cells to 80.22 ± 27.03% following 3 Gy UHDR irradiation (***p < 0.001). Non-irradiated U87MG ΔαBK_Ca_ cells showed 91.70 ± 10.54% head DNA, which did not differ significantly from non-irradiated U87MG wt cells (*p* = 0.7783). Irradiated U87MG ΔαBK_Ca_ cells showed 76.68 ± 30.60% head DNA (***p < 0.001 vs non-irradiated U87MG wt cells). Tail DNA correspondingly increased from 7.46 ± 8.81% in non-irradiated U87MG wt cells to 63.28 ± 32.65% following irradiation (***p < 0.001). Non-irradiated U87MG ΔαBK_Ca_ cells exhibited 14.36 ± 17.46% tail DNA, which was significantly higher than in non-irradiated U87MG wt cells (*p < 0.05). Following irradiation, U87MG ΔαBK_Ca_ cells showed a pronounced increase in tail DNA to 82.61 ± 19.80% (***p < 0.001 vs non-irradiated U87MG wt cells). Overall, the decrease in head DNA accompanied by the increase in tail DNA demonstrates pronounced DNA strand damage following UHDR irradiation.

### Cells lacking the BK_Ca_ channel exhibited reduced γH2AX accumulation following UHDR irradiation

Flow cytometry analysis of γH2AX enables quantitative assessment of DNA damage at the single-cell level and allows correlation with DNA content in large populations of cells. Therefore, γH2AX was used in our study as a marker of DNA double-strand breaks, detected using an Alexa Fluor 647-conjugated anti-H2AX (pS139) antibody (Fig. 4C–D). Under basal conditions, U87MG ΔαBK_Ca_ cells exhibited a lower percentage of γH2AX-positive cells compared with wt cells (29.83 ± 10.42% vs 45.53 ± 3.29%, *p ≤ 0.05). Positive control ETOP markedly increased γH2AX percentage in wt cells (78.00 ± 7.94%) compared with untreated controls (45.53 ± 3.29%, *p ≤ 0.05). 30 min after 3 Gy UHDR irradiation, the percentage of γH2AX-positive cells significantly increased in U87MG wt cells (69.47 ± 13.94%) compared with untreated U87MG wt controls (45.53 ± 3.29%; *p ≤ 0.05). In contrast, ΔαBK_Ca_ cells showed substantially lower γH2AX accumulation following IR (23.73 ± 5.57%) compared with wt cells (69.47 ± 13.94%, ***p ≤ 0.001). A comparable but less pronounced increase was observed in ΔαBK_Ca_ cells treated with ETOP (43.77 ± 6.17%), which did not differ significantly from untreated ΔαBK_Ca_ cells (ns).

### 53BP1 recruitment following UHDR irradiation in U87MG WT and U87MG ΔαBK_Ca_ cells

To further assess the DNA damage response following irradiation, 53BP1 recruitment was analyzed by immunofluorescence in U87MG wt and U87MG ΔαBK_Ca_ cells under control conditions and approximately 30 min after UHDR irradiation (3 Gy) (Fig. 4E–F). Etoposide (ETOP; 50 µM) was included as a positive control for DNA damage induction and is shown in the representative images (Fig. 4E). Under basal conditions, both cell lines exhibited relatively low numbers of 53BP1 foci. Following irradiation, U87MG wt cells showed a significant increase in the number of 53BP1 foci compared with non-irradiated wt cells (**p < 0.01). Importantly, irradiated U87MG ΔαBK_Ca_ cells exhibited significantly fewer 53BP1 foci than irradiated U87MG wt cells (***p < 0.001). (Fig. 4F). Total 53BP1 protein levels were additionally assessed by Western blot analysis (Fig. 4G–H). Densitometric analysis of three independent experiments, with 53BP1 band intensity normalized to β-actin, showed lower mean 53BP1/β-actin ratios in U87MG ΔαBK_Ca_ cells compared with wt cells under both control and irradiated conditions. However, the difference in 53BP1 protein levels between irradiated U87MG wt and U87MG ΔαBK_Ca_ cells was not statistically significant.

### BK_Ca_ deletion modulates the transcription of DNA repair genes after UHDR irradiation

**Fig. 5:**
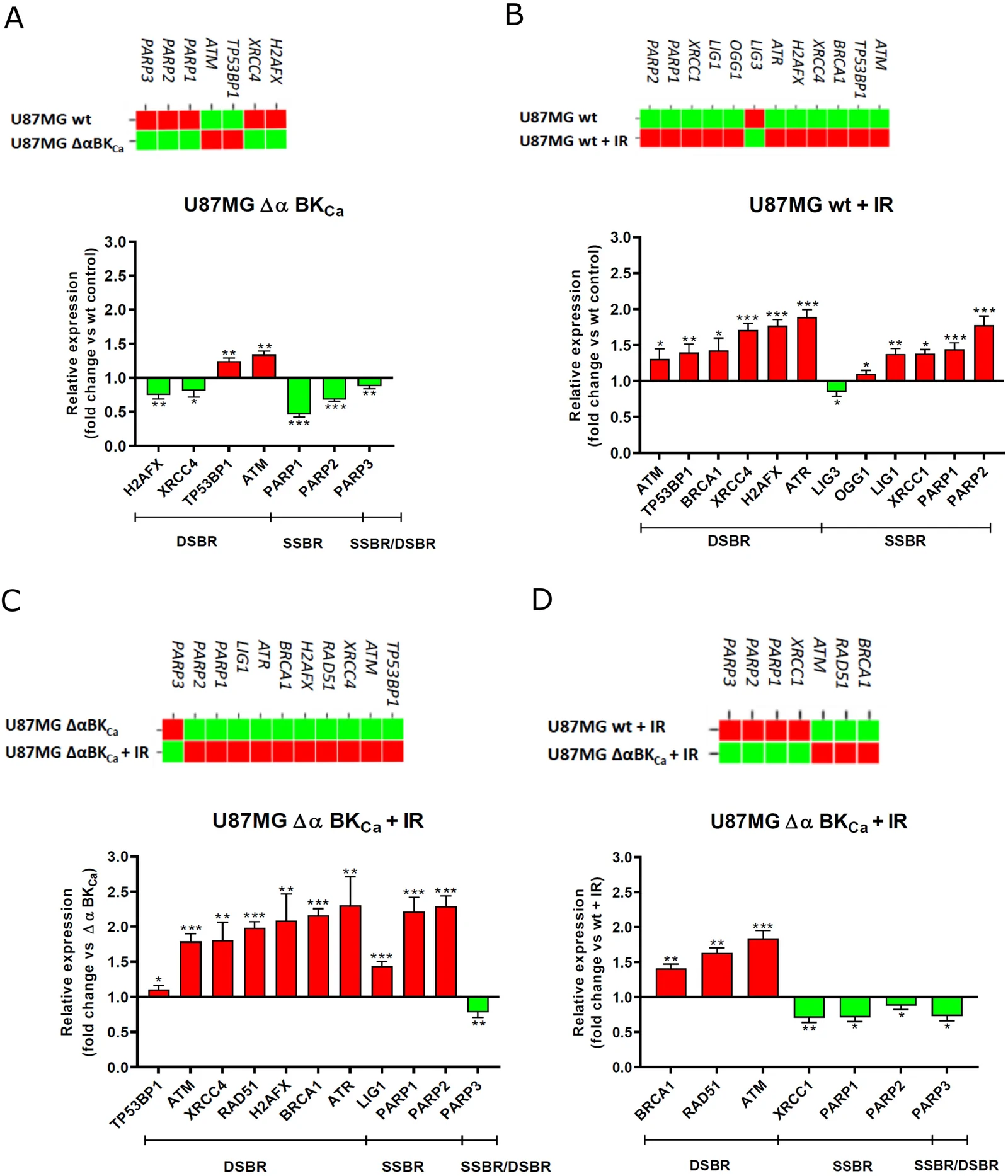
Analysis of DNA damage response gene expression in U87MG cells following UHDR irradiation. A: relative expression of DNA damage response genes in U87MG ΔαBK_Ca_ cells compared with U87MG wt cells under basal conditions. Expression levels are presented as Fold change relative to U87MG wt control cells. B: relative expression of DNA damage response genes in U87MG wt cells following IR. Expression levels are presented as Fold change relative to non-irradiated U87MG wt cells. C: relative expression of DNA damage response genes in U87MG ΔαBK_Ca_ cells following IR. Expression levels are presented as Fold change relative to non-irradiated U87MG ΔαBK_Ca_ cells. D: relative expression of selected DNA repair genes in U87MG ΔαBK_Ca_ cells compared with U87MG wt cells following IR. Expression levels are presented as Fold change relative to irradiated U87MG wt cells. Expression levels are presented as Fold change relative to the respective control conditions. Bars represent mean ± SD from three independent experiments (*n* = 3). Statistical analysis was performed using one-way analysis of variance (ANOVA). Statistical significance is indicated as *p ≤ 0.05, **p ≤ 0.01, ***p ≤ 0.001; ns – not significant. Above each graph, a summarized heatmap of gene expression changes is shown, with red indicating upregulation and green indicating downregulation relative to the corresponding control. The heatmaps show averaged values from the three independent experiments analyzed. The complete heatmap is presented in Supplementary Figure S2

mRNA levels of selected 14 genes (*ATM*, *ATR*, *BRCA1*, *H2AFX*, *LIG1*, *LIG3*, *OGG1*, *PARP1*, *PARP2*, *PARP3*, *RAD51*, *TP53BP1*, *XRCC1*, *XRCC4*) involved in double-strand break repair (DSBR) and single-strand break repair (SSBR) (Table S1) were analyzed in U87MG wt and U87MG ΔαBK_Ca_ cells under basal conditions and following irradiation (Fig. 5A–D, S2 and Table 2). The actin beta (*ACTB*) housekeeping gene was used as the reference gene. Under basal conditions (Fig. 5A), U87MG ΔαBK_Ca_ cells showed significantly reduced transcription of *H2AFX*, *XRCC4*, *PARP1*, *PARP2*, and *PARP3* compared with wt cells, while *TP53BP1* and *ATM* were upregulated. Irradiation of wt cells (Fig. 5B and S2) induced significant upregulation of multiple DSBR-related genes, including *ATM*, *TP53BP1*, *BRCA1*, *XRCC4,* and *H2AFX*, as well as SSBR-associated genes such as *LIG1*, *XRCC1*, *PARP1,* and *PARP2*. In ΔαBK_Ca_ cells exposed to IR (Fig. 5C and S2), a broad upregulation of DSBR genes was observed, including *TP53BP1*, *ATM*, *XRCC4*, *RAD51*, *H2AFX*, *BRCA1*, *ATR*, *LIG1*, *PARP1,* and *PARP2*. In contrast, *PARP3* mRNA levels were downregulated. Direct comparison of irradiated ΔαBK_Ca_ and irradiated wt cells (Fig. 5D and S2) revealed higher mRNA levels of *BRCA1*, *RAD51,* and *ATM* in ΔαBK_Ca_ cells, whereas *XRCC1*, *PARP1*, *PARP2,* and *PARP3* were downregulated relative to wt + IR. Significance levels are shown in the graphs.

**Table 2:** Summary of mRNA levels of tested DDR genes. ↓ downregulation, ↑ upregulation, - no changes

Gene	U87MG WT	**U87MG ΔαBK**_**Ca**_	U87MG WT + IR	**U87MG ΔαBK**_**Ca**_ **+ IR**	DDR pathway
*ATM*	**↓**	**↓**	**↓**	**↑**	DSBR
*ATR*	**↓**	**↓**	**↑**	**↑**	DSBR
*BRCA1*	**↓**	**↓**	**↑**	**↑**	DSBR
*H2AFX*	**↓**	**↓**	**↑**	**↑**	DSBR
*LIG1*	**↓**	**↓**	**↑**	**↑**	BER
*LIG3*	**↑**	**↓**	**↓**	**↑**	SSBR/BER
*OGG1*	**↓**	**↑**	**↓**	**↑**	SSBR/BER
*PARP1*	-	**↓**	**↑**	-	SSBR/BER
*PARP2*	**↓**	**↓**	**↑**	**↑**	SSBR/BER
*PARP3*	**↑**	-	**↑**	**↓**	SSBR/DSBR
*RAD51*	**↓**	**↓**	**↑**	**↑**	DSBR
*TP53BP1*	**↓**	-	**↑**	**↑**	DSBR
*XRCC1*	**↓**	**↓**	**↑**	**↓**	SSBR/BER
*XRCC4*	**↓**	**↓**	**↑**	**↑**	DSBR

## Discussion

In this study, we investigated the role of BK_Ca_ channel deletion in modulating glioblastoma cell response to UHDR, using clonogenic survival as a measure of long-term proliferative capacity. In radiobiological research, the examination of clonogenic survival and colony-forming capacity is regarded as the gold standard in vitro experimental endpoint [15]. Our results demonstrate a clear dose-dependent reduction in survival in both U87MG wt and U87MG ΔαBK_Ca_ cells following irradiation, confirming the expected cytotoxic effect of increasing radiation dose. The loss of clonogenic potential may arise both passively, as a consequence of unrepaired or incorrectly repaired DNA damage, and actively through the engagement of regulated cell death pathways, including apoptosis, governed by intracellular signaling networks [15, 42, 43]. Recent studies further emphasize that the radiation response of glioblastoma is strongly influenced by the capacity of tumor cells to activate and adapt DNA damage response and repair pathways, which represent major determinants of treatment resistance and potential targets for radiosensitization [44]. Therefore, we investigated the underlying mechanisms by assessing intracellular ROS levels, apoptosis, and DNA damage via γH2AX accumulation, DNA repair through 53BP1 foci formation, and the activation of DNA repair pathways at the level of transcriptional changes.

Cancer cells, including glioblastoma, may exhibit distinct responses to UHDR irradiation due to differences in redox homeostasis, oxygen availability, mitochondrial function, and DNA damage responses compared with normal cells [45]. Recent studies and reviews indicate that altered radical chemistry, oxidative stress, mitochondrial responses, and differential DNA damage processing remain among the major biological mechanisms proposed to contribute to cellular responses to UHDR irradiation, although their relative contribution remains unresolved [46, 47]. In our model, BK_Ca_-deficient cells exhibited higher ROS levels compared to wild-type cells following irradiation. Consistent with these findings, it has previously been demonstrated in the same cellular model that the absence of BK_Ca_ channels results in increased mitochondrial reactive oxygen species [25]. This suggests that BK_Ca_ channels may help maintain mitochondrial redox homeostasis and that their absence may render cells more vulnerable to increased oxidative stress when exposed to radiation. The increased oxidative stress observed in BK_Ca_-deficient cells may underlie alterations in DNA repair efficiency and cell cycle regulation.

The distinct cell-cycle responses observed following UHDR irradiation and etoposide treatment suggest that U87MG cells respond differently to different forms of genotoxic stress. Etoposide induced a predominantly G2/M-associated response in both cell lines, consistent with its established effects on DNA damage and cell-cycle checkpoint activation [48]. In contrast, following UHDR irradiation, U87MG ΔαBK_Ca_ cells showed a significant accumulation in the G0/G1 phase compared with irradiated U87MG wt cells, suggesting that BK_Ca_ deficiency modifies cell-cycle redistribution after irradiation. This observation is particularly relevant in the context of the other responses detected in BK_Ca_-deficient cells, including enhanced ROS accumulation and altered early DNA damage response signaling characterized by reduced γH2AX accumulation and 53BP1 recruitment. However, the present data do not establish a direct causal mechanism linking BK_Ca_ deletion to G0/G1 accumulation, and further studies examining specific checkpoint regulators will be required to define the molecular basis of this response.

UHDR irradiation promoted an early apoptotic response in both cell lines, as demonstrated by decreased cell viability and increased early apoptosis in the Annexin V/PI assay. However, neither viability nor early apoptosis differed significantly between irradiated U87MG wt and U87MG ΔαBK_Ca_ cells, indicating that BK_Ca_ deletion did not significantly modify the overall early apoptotic response under these conditions. Consistent with the induction of apoptotic signaling, the cleaved PARP1-positive fraction increased significantly after UHDR irradiation in U87MG ΔαBK_Ca_ cells, whereas the increase in U87MG wt cells did not reach statistical significance. Nevertheless, cleaved PARP1 levels did not differ significantly between the irradiated cell lines, and Western blot analysis likewise showed no genotype-dependent difference in the PARP1 cleavage ratio, arguing against a markedly enhanced apoptotic response caused by BK_Ca_ deletion. UHDR induced a markedly lower accumulation of γH2AX in BK_Ca_-deficient cells compared to wild-type cells, despite increased oxidative stress and apoptosis observed in this model. Importantly, the alkaline comet assay demonstrated pronounced DNA migration following UHDR irradiation, with decreased head DNA and increased tail DNA, confirming substantial DNA strand damage under the applied irradiation conditions. Thus, the reduced γH2AX accumulation and 53BP1 foci formation observed in BK_Ca_-deficient cells should not be interpreted as evidence of an absence of DNA damage, but rather as reflecting an altered early DNA damage response to UHDR irradiation. Therefore, we further assessed 53BP1 foci formation to determine whether DNA damage recognition and repair were also altered in these cells. 53BP1 is an early DNA damage response protein that binds to γH2AX-marked chromatin at sites of DSBs. It plays a key role in regulating the choice of DNA repair pathway, promoting nonhomologous end-joining while limiting homologous recombination, particularly in compact chromatin regions [15]. U87MG ΔαBK_Ca_ cells exhibited reduced 53BP1 foci formation following UHDR irradiation. These results suggest that BK_Ca_ deficiency may specifically alter the cellular response to UHDR irradiation and consequently affect DNA damage response signaling. This is consistent with the observed reduction in γH2AX accumulation and supports the notion of dysregulated DNA damage signaling in BK_Ca_-deficient cells. Importantly, the magnitude of early DNA damage marker accumulation does not necessarily reflect the overall extent of DNA damage or subsequent cellular outcome, as radiation responses are shaped by the temporal coordination of damage recognition, repair pathway engagement, replication stress, cell-cycle progression, and cell fate decisions. Recent studies in glioblastoma have highlighted the complexity and temporal dynamics of radiation-induced DNA damage responses, including the contribution of replication stress and ATR-dependent signaling to the survival of irradiated cells [49]. Previous studies have shown that while FLASH-RT does not significantly alter initial γH2AX foci formation, it can selectively reduce 53BP1-dependent DNA damage processing in normal cells, suggesting modulation of specific repair pathways rather than overall DSB induction [50].

Following UHDR irradiation, both cell lines exhibited transcriptional changes in DNA repair-related genes; however, U87MG ΔαBK_Ca_ cells showed a distinct profile characterized by higher expression of the DSBR-associated genes *BRCA1*, *RAD51*, and *ATM*, together with lower expression of the SSBR-associated genes *XRCC1*, *PARP1*, *PARP2*, and *PARP3* compared with irradiated U87MG wt cells. These transcriptional differences suggest altered regulation of the DNA damage response following BK_Ca_ deletion but should not be interpreted as direct evidence of functional activation or inhibition of the corresponding repair pathways. The potential relevance of altered ATM and homologous recombination signaling to glioblastoma radiation responses is supported by recent evidence identifying aberrant ATM signaling and homology-directed DNA repair as therapeutically exploitable features of irradiated GBM [51].

**Fig. 6:**
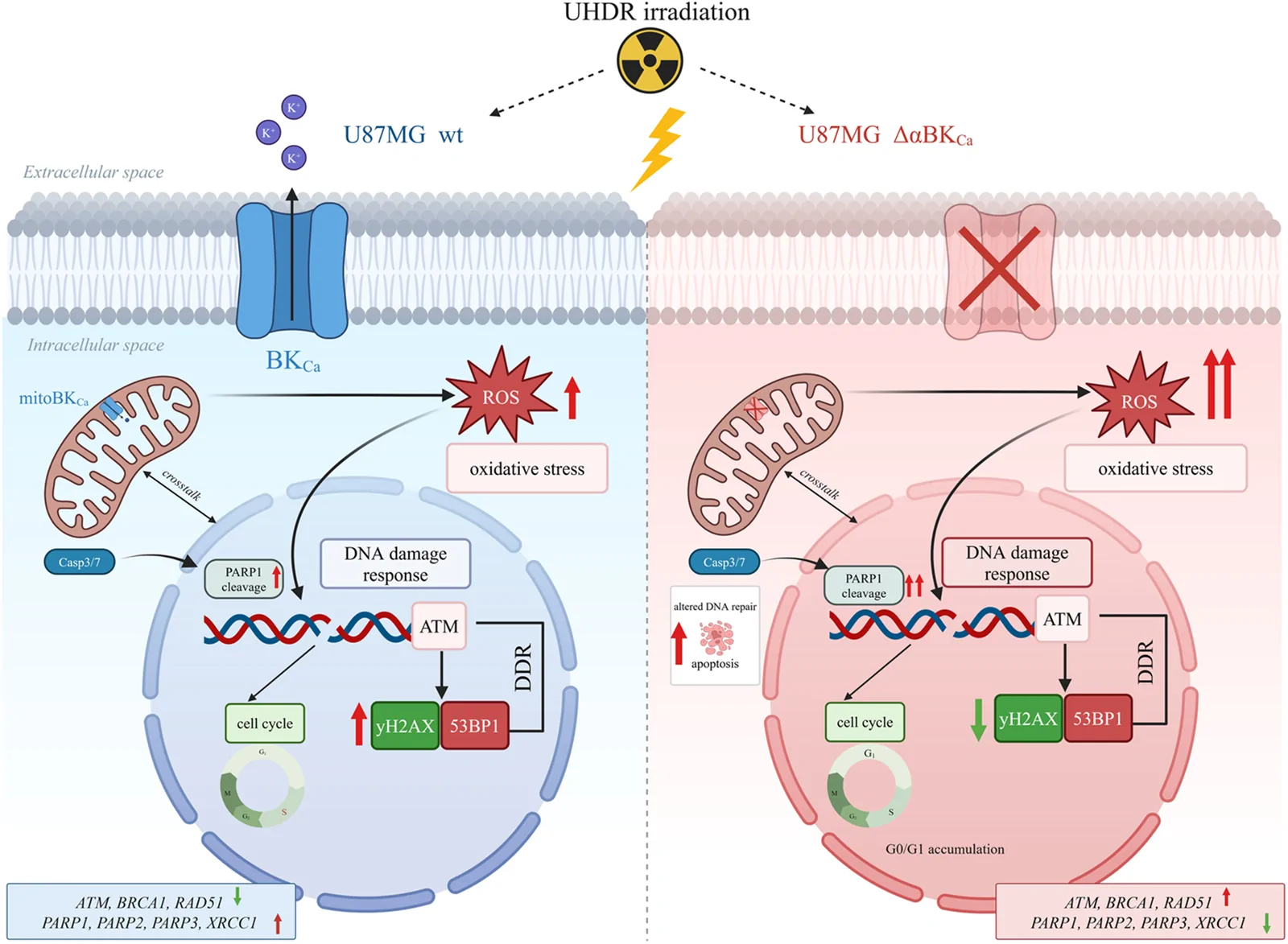
Proposed model of BK_Ca_-dependent modulation of the cellular response to UHDR irradiation in U87MG glioblastoma cells. Schematic representation of the cellular responses to UHDR irradiation in U87MG wt and U87MG ΔαBK_Ca_ cells. In U87MG wt cells, functional plasma membrane BK_Ca_ and mitochondrial BK_Ca_ (mitoBK_Ca_) channels are associated with lower ROS accumulation following irradiation. UHDR-induced DNA damage is accompanied by increased γH2AX accumulation and 53BP1 recruitment, consistent with activation of the early DNA damage response (DDR). In contrast, loss of BK_Ca_ in U87MG ΔαBK_Ca_ cells is associated with enhanced ROS generation and oxidative stress. Despite the presence of irradiation-induced DNA damage, BK_Ca_-deficient cells exhibit reduced γH2AX accumulation and 53BP1 recruitment compared with irradiated U87MG wt cells, indicating an altered early DDR. This response is accompanied by G0/G1 accumulation and increased PARP1 cleavage. Following UHDR irradiation, apoptosis increases in both cell lines, whereas the enhanced PARP1 cleavage observed in U87MG ΔαBK_Ca_ cells suggests modulation of the apoptotic response. qPCR analysis further indicates an altered DDR-related transcriptional profile in irradiated U87MG ΔαBK_Ca_ cells, with increased *ATM, BRCA1*, and *RAD51* and decreased *PARP1, PARP2, PARP3*, and *XRCC1* mRNA levels relative to irradiated U87MG wt cells. Arrows indicate increases or decreases relative to the corresponding comparison shown in the schematic. Created in *BioRender.com*

Recent evidence from orthotopic patient-derived glioblastoma xenografts further indicates that proton FLASH irradiation can achieve tumor control comparable to conventional proton radiotherapy while reducing DNA and RNA damage in surrounding normal brain tissue [52]. These findings highlight the potential relevance of UHDR approaches to glioblastoma treatment, but also emphasize the importance of direct UHDR-versus-conventional comparisons when attributing biological responses specifically to dose rate. In the present study, such a comparison was not performed; therefore, the observed effects cannot be considered specific to FLASH-RT. Taken together, our findings support a model in which BK_Ca_ deletion modulates the cellular response to UHDR irradiation by altering oxidative stress, early DNA damage response signaling, cell-cycle distribution, apoptotic signaling, and the transcriptional response of DNA repair-related genes, as summarized in Fig. 6.

Several limitations of the present study should be acknowledged. First, the experiments were performed using a single isogenic U87MG cell model, and therefore the findings should be validated in additional glioblastoma cell lines and, importantly, in patient-derived glioblastoma models. Second, the study was conducted exclusively in vitro, and the biological and potential translational relevance of the observed effects of BK_Ca_ deletion requires further validation in appropriate in vivo glioblastoma models. Third, the present study was designed to investigate the biological consequences of BK_Ca_ deletion under electron UHDR irradiation rather than to directly compare CONV-RT and FLASH-RT modalities. Consequently, the observed responses cannot be attributed specifically to FLASH-RT, and the conclusions should be restricted to the irradiation conditions investigated here. Further studies using complementary glioblastoma models, conventional dose-rate controls, and in vivo approaches will therefore be required to determine the broader biological and translational relevance of these findings. From a translational perspective, future studies should determine whether pharmacological modulation of BK_Ca_ could enhance the response of glioblastoma to radiotherapy in combination with temozolomide and current standard-of-care treatment, although the present findings do not provide evidence for BK_Ca_-dependent radiosensitization.

In summary, our findings identify BK_Ca_ as a potential modulator of the cellular response of glioblastoma cells to UHDR irradiation, associated with changes in oxidative stress, DNA damage response signaling, cell-cycle distribution, and apoptotic signaling. These observations provide a basis for further studies investigating whether BK_Ca_-dependent mechanisms could influence glioblastoma radiation responses and potentially represent a target for therapeutic modulation.

## Electronic supplementary material

Below is the link to the electronic supplementary material.

Supplementary Material 1

## Data Availability

All data generated or analyzed during the current study are included.
