# Supplementary material for: BKCa channel deletion modulates the DNA damage response to electron ultra-high dose rate irradiation in glioblastoma cells
Source: J Neurooncol. 2026 Sep 22;179(3):95. doi: 10.1007/s11060-026-05804-z (PMC13597620; doi:10.1007/s11060-026-05804-z)
Supplement: Supplementary file 1 [file 11060_2026_5804_MOESM1_ESM.pdf]

Figure S1

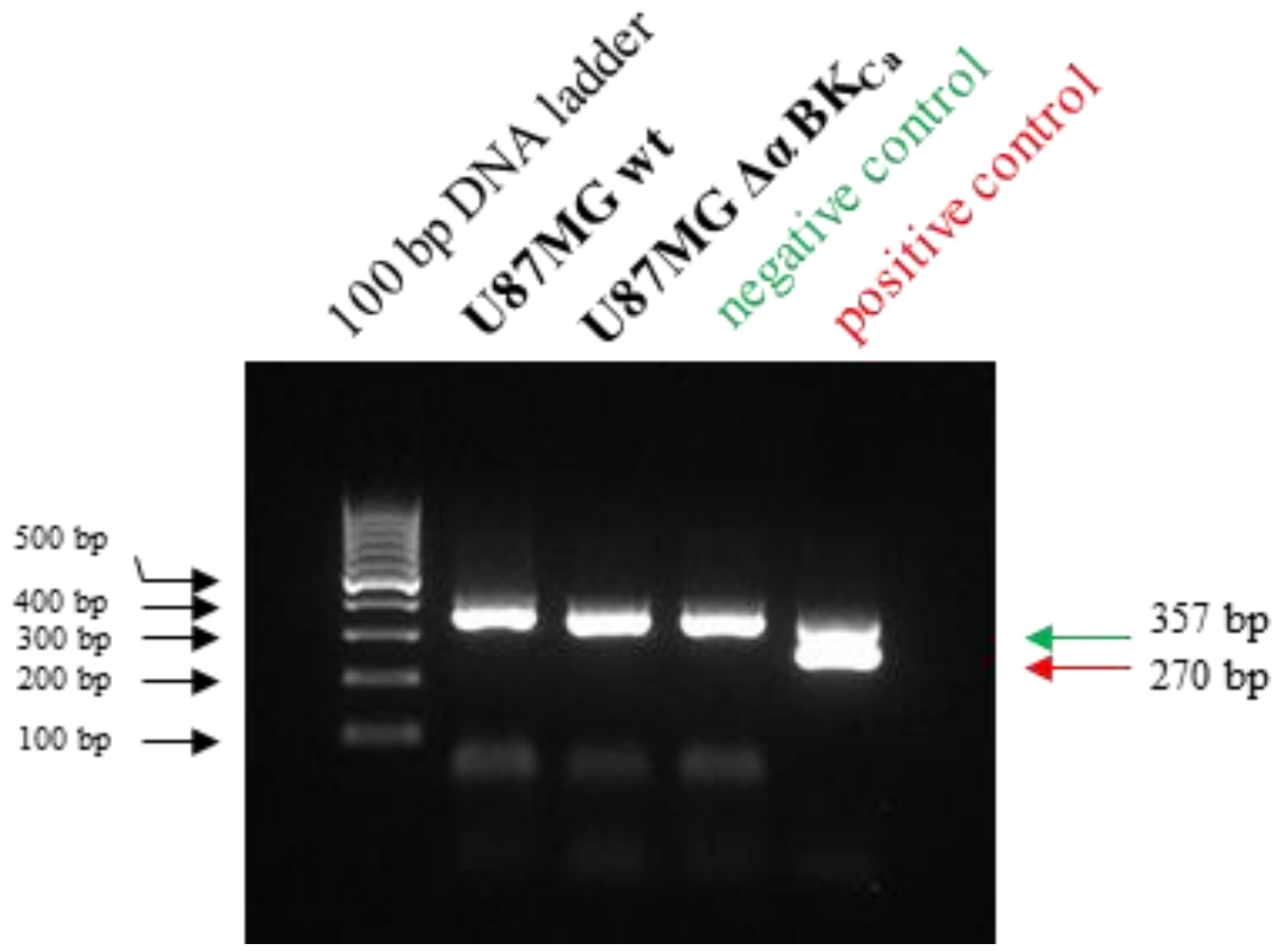

Figure S1. *Mycoplasma* test based on PCR using EZ-PCR Mycoplasma Detection Kit (Cat. No.: 20-700-20). Conditions: 10 µl PCR/lane, 2% agarose, 0.5 x TBE, 100V, 45 min. The *Mycoplasma* positive control shows a 270 bp band and a 357 bp band. *Mycoplasma*-negative samples (from U87MG wt and U87MG ΔαBK_{Ca} cell lines) and the negative control show a 357 bp band. Primer self-annealing yields bands of <100 bp. Ladder: 100 bp GeneRuler DNA ladder (ThermoFisher Scientific, SM0243). Original, uncropped gel.

Figure S2

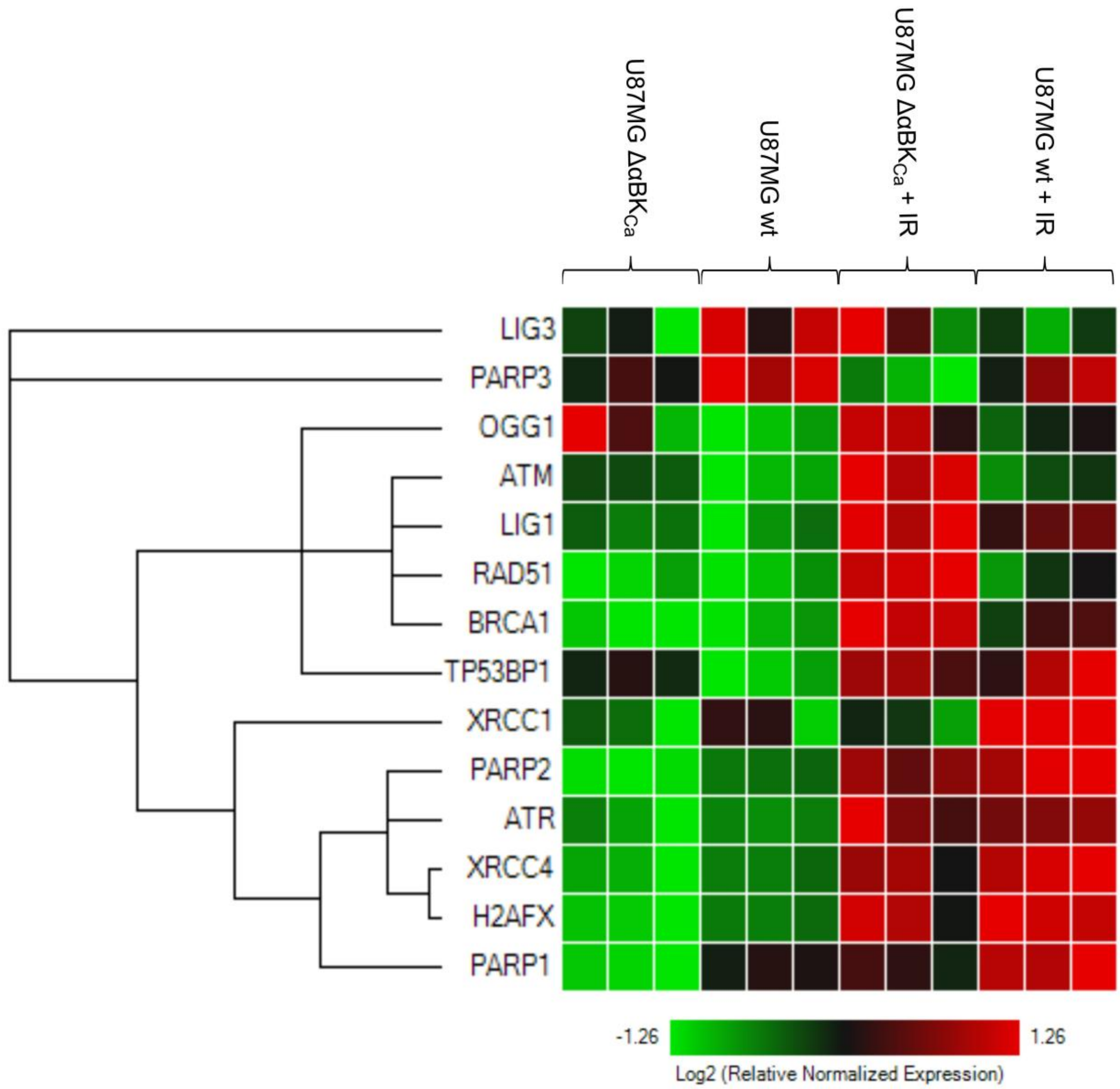

Figure S2. Combined cluster heatmap analysis of the normalized expression of DNA-damage signaling genes. U87MG wt, U87MG wt + IR, U87MG ΔαBK$_{Ca}$, U87MG ΔαBK$_{Ca}$ + IR. The red color characterizes a relatively high level of gene expression, whereas the green color indicates a low level. The data were analyzed (n=3) and clustered by targets using the Reference Gene Selection Tool from Bio-Rad CFX Maestro software.

Figure S3

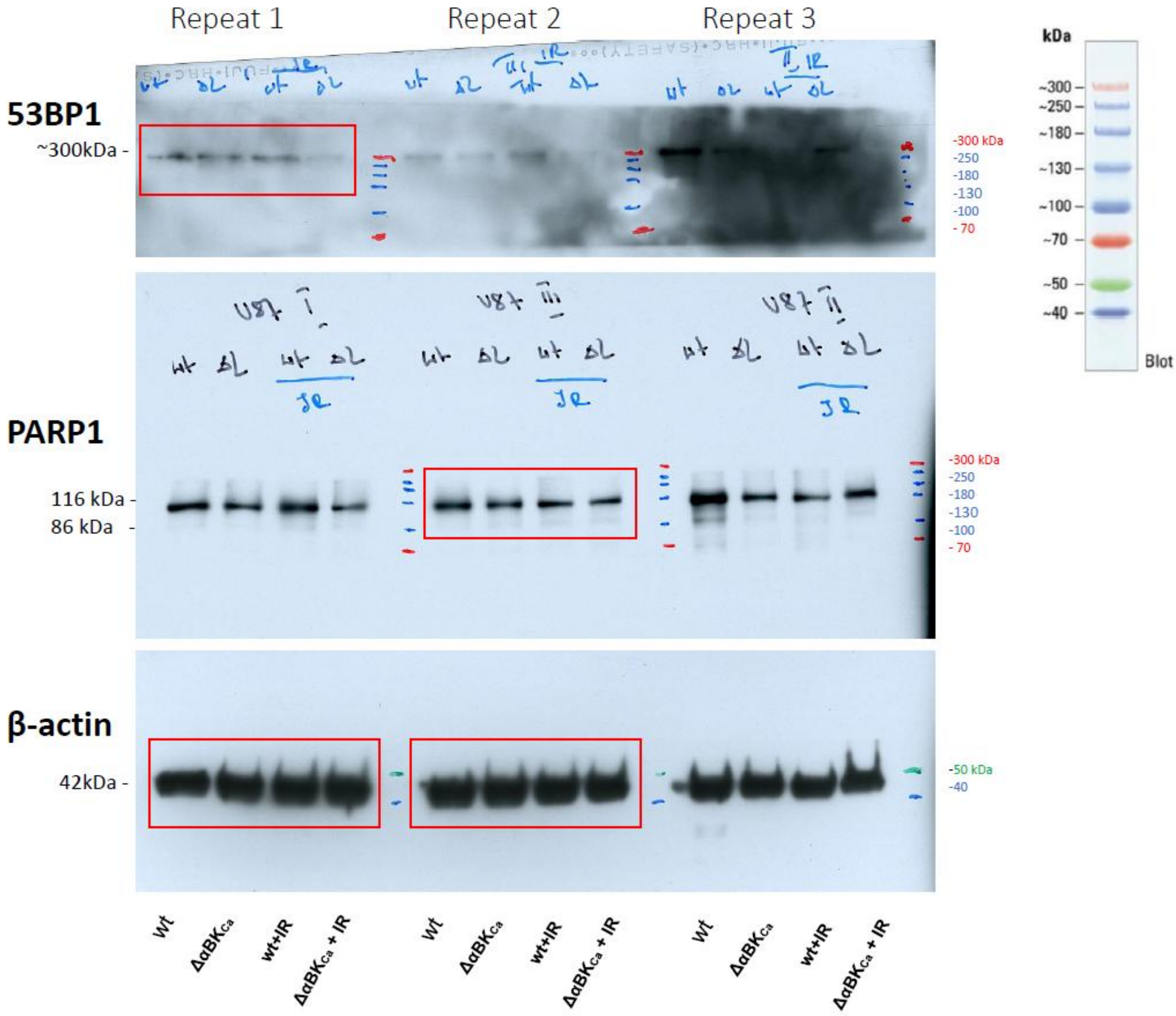

Figure S3. Original uncropped images of Western blots from three independent irradiation experiments used for the preparation of Figure 3E and Figure 4G. Human 53BP1 Antibody, R&D Systems, AF1877; Anti-PARP Polyclonal Antibody, ThermoFisher Scientific, PA5-34803: full-length PARP1 – 116 kDa, cleaved PARP1 – 89 kDa; Anti-β actin; Abcam ab49900. Samples from the left: U87MG wt, U87MG $\Delta\alpha BK_{Ca}$, U87MG wt + IR, U87MG $\Delta\alpha BK_{Ca}$ + IR. The red boxes indicate the bands that were selected for publication. Protein ladder: Spectra™ Multicolor High Range Protein Ladder, ThermoFisher Scientific, 26625.

Figure S4

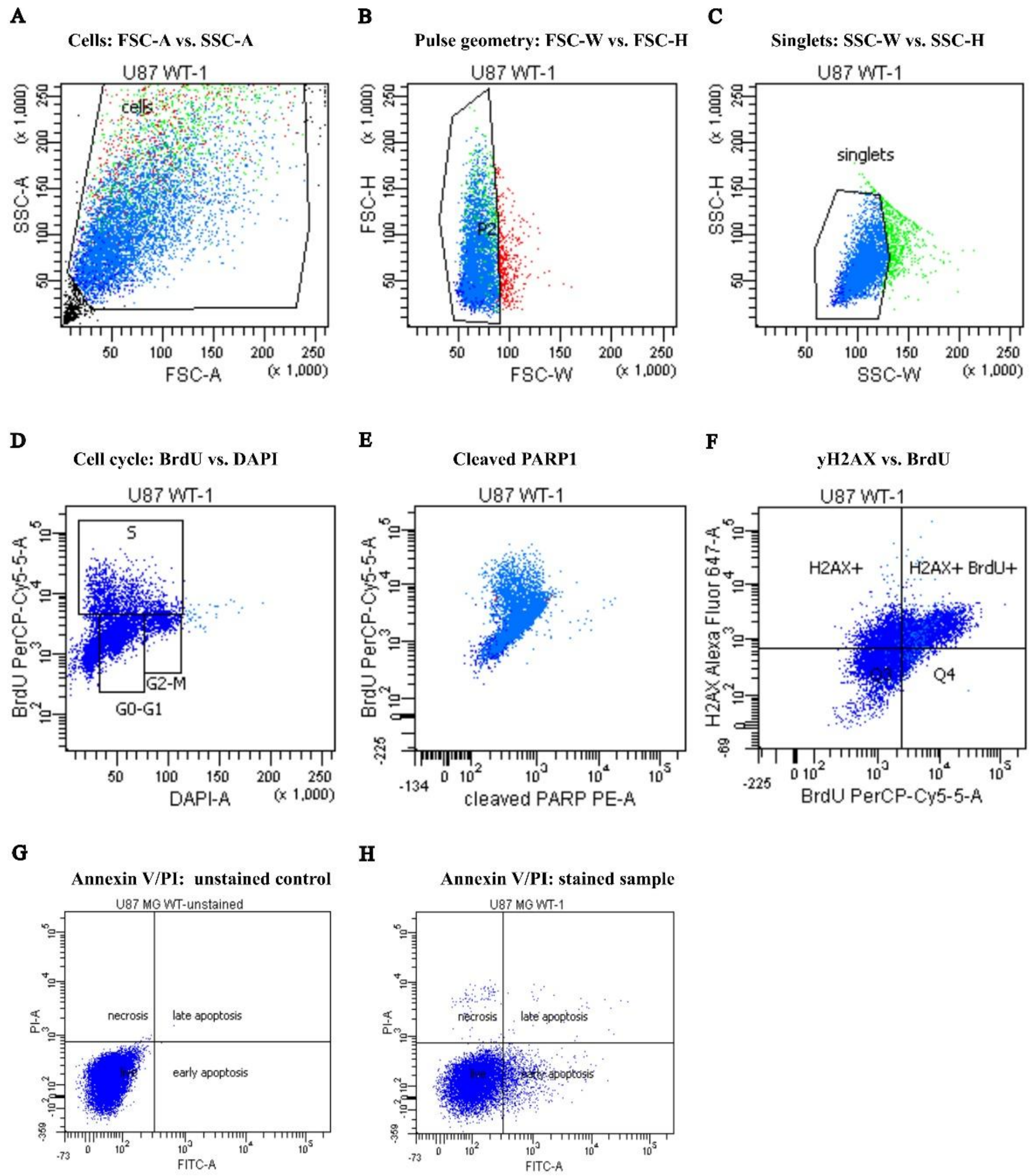

Figure S4. Representative flow cytometry gating strategy for cell-cycle, DNA-damage, cleaved PARP1, and Annexin V/PI apoptosis analyses. (A) The main cellular population was identified based on FSC-A/SSC-A characteristics, excluding events outside the cellular gate, including debris. (B–C) Sequential pulse-geometry gating based on FSC and SSC parameters was used for singlet discrimination and exclusion of cell aggregates/doublets. (D) Representative BrdU/DAPI plot used for cell-cycle analysis and discrimination of G0/G1, S, and G2/M populations. (E) Representative plot used for the analysis of cleaved PARP1-positive cells. (F) Representative γH2AX/BrdU plot used for the analysis of γH2AX-positive populations. (G) Representative unstained control used for Annexin V/PI analysis. (H) Representative Annexin V-FITC/PI plot showing classification of cells as viable (Annexin V−/PI−), early apoptotic (Annexin V+/PI−), late apoptotic (Annexin V+/PI+), and necrotic (Annexin V−/PI+) cells. The same assay-specific gating strategies were consistently applied to each cell line across the corresponding experimental conditions and biological replicates. The plots shown are representative examples of the gating strategy; quantitative analyses were performed using three independent biological experiments.

Supplementary Table S1.

The list of genes from selected DNA damage response (DDR) pathways with specific unique PrimePCR™Assay ID (Bio-Rad):

Gene	DDR pathway	Unique Bio-Rad PrimePCR™Assay ID
ATM	DNA double-strand breaks repair (DSBR)	qHsaCED0045448
ATR	DNA double-strand breaks repair (DSBR)	qHsaCID0022638
BRCA1	DNA double-strand breaks repair (DSBR)-HR	qHsaCED0038604
H2AFX	DNA double-strand breaks repair (DSBR)	qHsaCED0007023
LIG1	the base excision repair (BER)	qHsaCID0008449
LIG3	single-strand breaks repair (SSBR)/BER	qHsaCID0018427
OGG1	single-strand breaks repair (SSBR)/BER	qHsaCID0012607
PARP1	single-strand breaks repair (SSBR)/BER	qHsaCED0045162
PARP2	single-strand breaks repair (SSBR)/BER	qHsaCED0044268
PARP3	single and double-strand breaks repair (SSBR/DSBR)	qHsaCED0002085
RAD51	DNA double-strand breaks repair (DSBR)-HR	qHsaCID0007576
TP53BP1	DNA double-strand breaks repair (DSBR)-NHEJ	qHsaCID0020917
XRCC1	single-strand breaks repair (SSBR)/BER	qHsaCID0023107
XRCC4	DNA double-strand breaks repair (DSBR)-NHEJ	qHsaCID0016093
